# Finite Element Iterative Methods for the Stationary Double-Diffusive Natural Convection Model

**DOI:** 10.3390/e24020236

**Published:** 2022-02-03

**Authors:** Yaxin Wei, Pengzhan Huang

**Affiliations:** College of Mathematics and System Sciences, Xinjiang University, Urumqi 830017, China; mathmxl115@xju.edu.cn

**Keywords:** double-diffusive natural convection, finite element discretization, iterative methods, viscosity, uniqueness condition

## Abstract

In this paper, we consider the stationary double-diffusive natural convection model, which can model heat and mass transfer phenomena. Based on the fixed point theorem, the existence and uniqueness of the considered model are proved. Moreover, we design three finite element iterative methods for the considered problem. Under the uniqueness condition of a weak solution, iterative method I is stable. Compared with iterative method I, iterative method II is stable with a stronger condition. Moreover, iterative method III is stable with the strongest condition. From the perspective of viscosity, iterative method I displays well in the case of a low viscosity number, iterative method II runs well with slightly low viscosity, and iterative method III can deal with high viscosity. Finally, some numerical experiments are presented for testing the correctness of the theoretic analysis.

## 1. Introduction

The double-diffusive natural convection model, which does not only incorporate the velocity vector field as well as the pressure field, but also contains the temperature field and the concentration field, has been widely used in scientific, engineering and industrial applications such as nuclear design, cooling of electronic equipment, aircraft cabins, insulation with double pane windows, and so on. For greater understanding of the physical background, authors can refer to [1,2,3]. In recent years, the impact of nanofluid on free convection heat transfer was investigated by researchers in [4]. The free convective flow of a Nano-Encapsulated Phase Change Material (NEPCM) suspension in an eccentric annulus was investigated numerically in [5]. The authors obtained that the volume fraction of the NEPCM particles and Stefan number effect the thermal and hydrodynamic characteristics of the suspension. The effect of the arrangement of the tubes in a multi-tube heat exchanger during the solidification process was considered in [6], which focused on the natural convection effect in phase change material in this research.

Let $\Omega \subset R^{2}$ be a open bounded domain with a Lipschitz continuous boundary ∂Ω and ∂Γ is a subset of ∂Ω, $u=(u_{1},u_{2})$ denotes the velocity field, *p* is the fluid pressure, *T* is the temperature, *C* is the concentration, g=(0,1) is the gravitational acceleration vector, $f_{i}$ is the forcing function, i=1,2. Moreover, n represents the outer normal vector, ν>0 is the viscosity, $D_{a}$ is the Darcy number, γ>0 is the heat diffusivity, $D_{c}$ is the mass diffusivity, $\beta_{T}$ and $\beta_{C}$ are the thermal and solutal expansion coefficients.

The governing equations of this double-diffusive natural convection model are presented as follows [7].

(1) $$\left\{\begin{array}{cccc} -\nu \left(\frac{\partial^{2}u_{1}}{\partial x^{2}}+\frac{\partial^{2}u_{1}}{\partial y^{2}}\right)+\left(u_{1}\frac{\partial u_{1}}{\partial x}+u_{2}\frac{\partial u_{1}}{\partial y}\right) & = & -D_{a}^{-1}u_{1}-\frac{\partial p}{\partial x}, & \mathrm{in}\;\;\Omega ,\\ -\nu \left(\frac{\partial^{2}u_{2}}{\partial x^{2}}+\frac{\partial^{2}u_{2}}{\partial y^{2}}\right)+\left(u_{1}\frac{\partial u_{2}}{\partial x}+u_{2}\frac{\partial u_{2}}{\partial y}\right) & = & \beta_{T}T+\beta_{C}C-D_{a}^{-1}u_{2}-\frac{\partial p}{\partial y}, & \mathrm{in}\;\;\Omega ,\\ \frac{\partial u_{1}}{\partial x}+\frac{\partial u_{2}}{\partial y} & = & 0, & \mathrm{in}\;\;\Omega ,\\ -\gamma \left(\frac{\partial^{2}T}{\partial x^{2}}+\frac{\partial^{2}T}{\partial y^{2}}\right)+\left(u_{1}\frac{\partial T}{\partial x}+u_{2}\frac{\partial T}{\partial y}\right) & = & f_{2} & \mathrm{in}\;\;\Omega ,\\ -D_{c}\left(\frac{\partial^{2}C}{\partial x^{2}}+\frac{\partial^{2}C}{\partial y^{2}}\right)+\left(u_{1}\frac{\partial T}{\partial x}+u_{2}\frac{\partial T}{\partial y}\right) & = & f_{2} & \mathrm{in}\;\;\Omega ,\\ u & = & 0, & \mathrm{on}\;\;\partial \Omega ,\\ T & = & 0,\;\;C\;=\;0, & \mathrm{on}\;\;\partial \Gamma ,\\ \frac{\partial T}{\partial n} & = & \frac{\partial C}{\partial n}\;=\;0, & \mathrm{on}\;\;\partial \Omega \backslash \partial \Gamma . \end{array}\right.$$

Many numerical studies were made concerning the double-diffusive natural convection model. A projection-based stabilized finite element method for steady-state natural convection problem was considered in [8]. A stabilized finite element error analysis for the Darcy–Brinkman model of double-diffusive convection in a porous medium was discussed in [9]. An efficient two-step algorithm for the steady-state natural convection problem was presented in [10]. The melting process of a nano-enhanced phase change material in a square cavity was investigated in [11]. In numerical test, the author used the Galerkin finite element method to solve the dimensionless partial differential equations. Based on the idea of curvature stabilization, Çıbık et al. [12] discussed a family of second order time stepping methods for the Darcy–Brinkman equations. A decoupled finite element method called the modified characteristics method was considered in [13]. Rajabi et al. performed the detailed uncertainty propagation analysis and variance-based global sensitivity analysis on the widely adopted double-diffuse convection benchmark problem of a square porous cavity with horizontal temperature and concentration gradients in [14]. In [15], the mixed convection heat transfer of AL2O3 nanofuid in a horizontal channel subjected with two heat sources was considered. In [16], the curvature-based stabilization method was considered for double-diffusive natural convection flows in the presence of a magnetic field and unconditionally stable and optimally accurate second order approximations were obtained. There are several works devoted to the efficient numerical methods for the treatment of nonlinear problems. For example, several iterative methods for the 2D steady penalty Navier–Stokes equations were presented and discussed in [17]. He et al. [18] discussed a combination of two-level methods and iterative methods for solving the 2D/3D steady Navier–Stokes equations. Some iterative finite element methods for steady Navier–Stokes equations with different viscosities were discussed in [19]. Furthermore, the authors refer to the Oseen method [20], the Newton method [21] and the Euler implicit-explicit methods [22]. Recently, Huang et al. [23] have considered and analyzed the Oseen, Newton and Stokes iterative methods for the 2D steady Navier–Stokes equations. He et al. [24] considered and analyzed three iterative methods for the 3D steady MHD equations.

The main work in this paper is to design, analyze, and compare three iteration methods to solve nonlinear equations based on the finite element discretization. Then, we will show the performance of these numerical methods in both theoretical analysis and numerical experiments. By setting $\sigma =m\alpha^{2}\nu^{-2}N\left(\gamma^{-1}{∥f_{1}∥}_{-1}+D_{c}^{-1}{∥f_{2}∥}_{-1}\right)+m\alpha^{2}\nu^{-1}\bar{N}\left(\gamma^{-2}{∥f_{1}∥}_{-1}+D_{c}^{-2}{∥f_{2}∥}_{-1}\right)$, we obtain the conclusion that the three iterative methods are stable and convergent as $\sigma \in (0,\frac{1}{4})$. Iterative method I and II are valid as $\sigma \in [\frac{1}{4},\frac{1}{3})$ and only iterative method I runs well as $\sigma \in [\frac{1}{3},1)$.

In this paper, by developing some techniques and using some ideas in [7], we prove the existence and uniqueness with a different method, then we obtain a different uniqueness condition. Furthermore, we propose and analyze iterative methods I and III. In addition to this, we use iterative method II to computer a smaller viscosity than them in numerical experiments. Compared with He et al. [24], although the iterative methods are the same, the considered problems are different.

The paper is organized as follows. In Section 2, we describe the considered problem and some mathematical preliminaries. In the next section, we prove the existence and uniqueness of the weak solution to the considered equations. Then, we analyze stability and iterative error estimates of three iterative methods in Section 4. In Section 5, we show some numerical experiments to verify the correctness of theoretical results. In the last section, conclusions are presented.

## 2. Preliminaries

In this section, we present some basic notations and properties for the stationary double-diffusive natural convection problem. First, for 1≤q≤∞ and $m\in N^{+},$ we use standard notations for Sobolev space $W^{m,q}\left(\Omega \right)$ and Lebegue space $L^{q}\left(\Omega \right)$. In particular, $L^{2}\left(\Omega \right)$ norm and its inner product are denoted by ${∥\cdot ∥}_{0}$ and (·,·). Moreover, for *f*, an element in the dual space of $H^{1}\left(\Omega \right)$, its norm is defined by

$${∥f∥}_{-1}=\underset{\psi \in H^{1}\left(\Omega \right)}{\sup}\frac{\left|(f,\psi )\right|}{{∥\nabla \psi ∥}_{0}}.$$

Next, we introduce the functional spaces associated with the velocity, the pressure, the temperature, and the concentration:

$$\begin{array}{cc} & X=\left\{u\in H^{1}{\left(\Omega \right)}^{2}:u{|}_{\partial \Omega }=0\right\},\;W=\left\{\psi \in H^{1}\left(\Omega \right):\psi {|}_{\partial \Gamma }=0\right\},\\ & Q=\left\{s\in H^{1}\left(\Omega \right):s{|}_{\partial \Gamma }=0\right\},\;M=\left\{q\in L^{2}\left(\Omega \right):\int_{\Omega }qdx=0\right\}. \end{array}$$

Then, we define the following particular subspace of the velocity space X

$$V=\left\{v\in X:\int_{\Omega }q\mathrm{div}\;vd\Omega =0,\forall q\in M\right\}.$$

Moreover, define several continuous bilinear forms $a_{0}\left(\cdot ,\cdot \right),$$a_{1}\left(\cdot ,\cdot \right),$$a_{2}\left(\cdot ,\cdot \right)$ and d(·,·) on X×X,W×W,Q×Q and X×M, respectively,

$$\begin{array}{cc} a_{0}\left(u,v\right) & =\nu \left(\nabla u,\nabla v\right),\;\;\forall u,v\in X,\;a_{1}\left(T,\psi \right)=\gamma \left(\nabla T,\nabla \psi \right),\;\forall T,\psi \in W,\\ a_{2}\left(C,s\right) & =D_{c}\left(\nabla C,\nabla s\right),\;\;\forall C,s\in Q,\;d\left(q,v\right)=\left(q,\mathrm{div}\;v\right),\;\;\forall v\in X,\forall q\in M. \end{array}$$

Further, denote three skew-symmetric trilinear forms:

$$\begin{array}{cc} c_{0}\left(u,v,w\right) & =\left(\left(u\cdot \nabla \right)v,w\right)+\frac{1}{2}\left(\left(\mathrm{div}\;u\right)v,w\right)\\ & =\frac{1}{2}\left(\left(u\cdot \nabla \right)v,w\right)-\frac{1}{2}\left(\left(u\cdot \nabla \right)w,v\right),\;\;\forall u,w,v\in X,\\ c_{1}\left(u,T,\psi \right) & =\left(\left(u\cdot \nabla \right)T,\psi \right)+\frac{1}{2}\left(\left(\mathrm{div}\;u\right)T,\psi \right)\\ & =\frac{1}{2}\left(\left(u\cdot \nabla \right)T,\psi \right)-\frac{1}{2}\left(\left(u\cdot \nabla \right)\psi ,T\right),\;\;\forall u\in X,\;\;T,\psi \in W,\\ c_{2}\left(u,C,s\right) & =\left(\left(u\cdot \nabla \right)C,s\right)+\frac{1}{2}\left(\left(\mathrm{div}\;u\right)C,s\right)\\ & =\frac{1}{2}\left(\left(u\cdot \nabla \right)C,s\right)-\frac{1}{2}\left(\left(u\cdot \nabla \right)s,C\right),\;\;\;\forall u\in X,\;\;C,s\in Q. \end{array}$$

Please note that the bilinear form d(·,·) is continuous on X×M and satisfies the inf-sup condition [25]: there exists a positive constant $\beta_{0}$ such that

$$\underset{v\in V}{\sup}\frac{\left|d(q,v)\right|}{{∥\nabla v∥}_{0}}\geq \beta_{0}{∥q∥}_{0},\;\forall q\in M.$$

The trilinear forms [18] satisfy

(2) $$\begin{array}{c} \begin{array}{cc} & c_{0}\left(u,v,w\right)=-c_{0}\left(u,w,v\right),\;\left|c_{0}\left(u,v,w\right)\right|\leq N_{0}{∥\nabla u∥}_{0}{∥\nabla v∥}_{0}{∥\nabla w∥}_{0}, \end{array} \end{array}$$

and

(3) $$\begin{array}{c} \begin{array}{cc} & c_{1}\left(u,T,\psi \right)=-c_{1}\left(u,\psi ,T\right),\;c_{2}\left(u,C,s\right)\;=-c_{2}\left(u,s,C\right),\\ & \left|c_{1}\left(u,T,\psi \right)\right|\leq N_{1}{∥\nabla u∥}_{0}{∥\nabla T∥}_{0}{∥\nabla \psi ∥}_{0},\;\left|c_{2}\left(u,C,s\right)\right|\leq N_{2}{∥\nabla u∥}_{0}{∥\nabla C∥}_{0}{∥\nabla s∥}_{0}, \end{array} \end{array}$$

where $N_{i}>0,$i=0,1,2, are three constants defined as $N_{0}=\sup_{u,v,w\in X}\frac{\left|c_{0}\left(u,v,w\right)\right|}{{∥\nabla u∥}_{0}{∥\nabla v∥}_{0}{∥\nabla w∥}_{0}},$ $N_{1}=\sup_{u\in X,T,\psi \in W}\frac{\left|c_{1}\left(u,T,\psi \right)\right|}{{∥\nabla u∥}_{0}{∥\nabla T∥}_{0}{∥\nabla \psi ∥}_{0}},$ and $N_{2}=\sup_{u\in X,C,s\in Q}\frac{\left|c_{2}\left(u,C,s\right)\right|}{{∥\nabla u∥}_{0}{∥\nabla C∥}_{0}{∥\nabla s∥}_{0}}.$

Furthermore, we recall the Poincaré inequality [25]

(4) $$\begin{array}{c} \begin{array}{c} {∥u∥}_{0}\leq \alpha {∥\nabla u∥}_{0},\;\forall u\in H^{1}\left(\Omega \right), \end{array} \end{array}$$

where α is a positive constant depending on Ω.

The variational form of the model (1) is presented as follows: find (u,p,T,C)∈X×M×W×Q such that for all (v,q,ψ,s)∈X×M×W×Q

(5) $$\left\{\begin{array}{c} a_{0}\left(u,v\right)+c_{0}\left(u,u,v\right)+D_{a}^{-1}\left(u,v\right)-d\left(p,v\right)+d\left(q,u\right)=\left(\beta_{T}Tg+\beta_{C}Cg,v\right),\\ a_{1}\left(T,\psi \right)+c_{1}\left(u,T,\psi \right)=\left(f_{1},\psi \right),\\ a_{2}\left(C,s\right)+c_{2}\left(u,C,s\right)=\left(f_{2},s\right). \end{array}\right.$$

## 3. Existence and Uniqueness

This section gives the existence and uniqueness of (5), which is crucial to consider the discrete scheme.

**Theorem** **1.** *There exists at least a solution pair (u,p,T,C)∈X×M×W×Q which satisfies* (5) *and*

(6) $$\begin{array}{c} \begin{array}{cc} {∥\nabla T∥}_{0} & \leq \gamma^{-1}{∥f_{1}∥}_{-1},\;{∥\nabla C∥}_{0}\leq D_{c}^{-1}{∥f_{2}∥}_{-1},\\ {∥\nabla u∥}_{0} & \leq \nu^{-1}m\alpha^{2}\left(\gamma^{-1}{∥f_{1}∥}_{-1}+D_{c}^{-1}{∥f_{2}∥}_{-1}\right). \end{array} \end{array}$$

**Proof**.  First, for u∈X, it is easy to see that $a_{1}\left(\cdot ,\cdot \right)+c_{1}\left(u,\cdot ,\cdot \right)$ and $a_{2}\left(\cdot ,\cdot \right)+c_{2}\left(u,\cdot ,\cdot \right)$ are continuous, elliptic bilinear forms of W×W and Q×Q, respectively. Hence, according to the Lax–Milgram theorem, there exists a unique solution T∈W to the second equation of (5), and a unique solution C∈Q to the third equation of (5). The theorem will be proved if we can show that there is at least a solution u∈X in the first equation of (5).

Secondly, $a_{0}\left(\cdot ,\cdot \right)$ is a continuous and elliptic bilinear form on X×X. Using (2) and (4) we obtain

$$\begin{array}{c} \left|-c_{0}\left(u,u,v\right)+\left(\beta_{T}Tg+\beta_{C}Cg,v\right)\right|\leq \left(N_{0}{∥\nabla u∥}_{0}^{2}+m\alpha^{2}\left({∥\nabla T∥}_{0}+{∥\nabla C∥}_{0}\right)\right){∥\nabla v∥}_{0}, \end{array}$$

where $m=\left|g\right|\max\{\left|\beta_{T}\right|,\left|\beta_{C}\right|\}$. Then, we define a mapping A:X→X by $A\left(u\right)=w_{1}$ where

(7) $$a_{0}\left(w_{1},v\right)+D_{a}^{-1}\left(w_{1},v\right)=-c_{0}\left(u,u,v\right)+\left(\beta_{T}Tg+\beta_{C}Cg,v\right),\;\forall v\in V.$$

Clearly, u is a solution of the first equation of (5) with v∈V, if it is a solution of A(u)=u. Using the Leray-Schauder Principle [26], A(u)=u has at least one solution u∈X, if (a) A is completely continuous; (b) there exists $M_{1}>0$ such that for every λ∈[0,1] and v∈X with λAv=v,v satisfies the bound ${∥\nabla v∥}_{0}\leq M_{1}$.

Assume $u_{1},$$u_{2}$∈X and subtract the equations obtained from (7) with $u=u_{1}$ and $u=u_{2}$. Then, set $w=A\left(u_{2}\right)-A\left(u_{1}\right)$ and choose v=w to yield

(8) $$\begin{array}{cc} a_{0}\left(w,w\right)+D_{a}^{-1}\left(w,w\right)=\; & -c_{0}\left(u_{2}-u_{1},u_{2},w\right)-c_{0}\left(u_{1},u_{2}-u_{1},w\right)\\ \; & +(\beta_{T}\left(T_{2}-T_{1}\right)g+\beta_{C}\left(C_{2}-C_{1}\right)g,w). \end{array}$$

Moreover, in order to estimate ${∥\nabla (T_{2}-T_{1})∥}_{0}$ and ${∥\nabla (C_{2}-C_{1})∥}_{0}$, we substitute $T_{1}$ and $T_{2}$ in the second equation of (5) and subtract the ensuing equations to obtain

$$a_{1}\left(T_{2}-T_{1},\psi \right)=-c_{1}\left(u_{2}-u_{1},T_{2},\psi \right)-c_{1}\left(u_{1},T_{2}-T_{1},\psi \right).$$

Taking $\psi =T_{2}-T_{1}$ and using (3) we obtain

(9) $${∥\nabla (T_{2}-T_{1})∥}_{0}\leq \gamma^{-1}N_{1}{∥\nabla (u_{2}-u_{1})∥}_{0}{∥\nabla T_{2}∥}_{0}.$$

Analogously, we have

(10) $${∥\nabla (C_{2}-C_{1})∥}_{0}\leq D_{c}^{-1}N_{2}{∥\nabla (u_{2}-u_{1})∥}_{0}{∥\nabla C_{2}∥}_{0}.$$

Further, combining (9) and (10), we obtain the bound of (8) as follows

$${∥\nabla w∥}_{0}\leq \nu^{-1}\left(N_{0}{∥\nabla u_{1}∥}_{0}+N_{0}{∥\nabla u_{2}∥}_{0}+m\alpha^{2}\left(\gamma^{-1}N_{1}{∥\nabla T_{2}∥}_{0}+D_{c}^{-1}N_{2}{∥\nabla C_{2}∥}_{0}\right)\right){∥\nabla (u_{2}-u_{1})∥}_{0}.$$

Hence, *A* is completely continuous.

Now, we prove (b). If λ=0, then v=0 and ${∥\nabla v∥}_{0}=0$. Assume λ∈(0,1] and v∈X satisfies λAv=v. Then, from (7), we have

$$\lambda^{-1}a_{0}\left(v,v\right)+\lambda^{-1}D_{a}^{-1}\left(v,v\right)=-c_{0}\left(v,v,v\right)+\left(\beta_{T}Tg+\beta_{C}Cg,v\right).$$

Using (2) and (4), we arrive at

$${∥\nabla v∥}_{0}\leq \nu^{-1}\lambda m\alpha^{2}\left({∥\nabla T∥}_{0}+{∥\nabla C∥}_{0}\right).$$

Thirdly, setting ψ=T in the second equation of (5), we have

$$\gamma {∥\nabla T∥}_{0}^{2}+c_{1}\left(u,T,T\right)\leq {∥f_{1}∥}_{-1}{∥\nabla T∥}_{0}.$$

Thus, applying (3) leads to

$${∥\nabla T∥}_{0}\leq \gamma^{-1}{∥f_{1}∥}_{-1}.$$

Similarly, taking s=C in the third equation of (5), we obtain

$${∥\nabla C∥}_{0}\leq D_{c}^{-1}{∥f_{2}∥}_{-1}.$$

Moreover, choosing v=u in the first equation of (5) and using (4), we arrive at

$$\nu {∥\nabla u∥}_{0}^{2}+c_{0}\left(u,u,u\right)\leq m\alpha^{2}\left({∥\nabla T∥}_{0}+{∥\nabla C∥}_{0}\right){∥\nabla u∥}_{0},$$

which combines with the above two equations to give

$${∥\nabla u∥}_{0}\leq \nu^{-1}m\alpha^{2}\left(\gamma^{-1}{∥f_{1}∥}_{-1}+D_{c}^{-1}{∥f_{2}∥}_{-1}\right).$$

The proof is completed. □

**Theorem** **2.** *Assume that (u,p,T,C)∈X×M×W×Q is a solution pair of (5). If ν, $D_{c}$, γ, C and T satisfy the following uniqueness condition*

$$\begin{array}{c} 0<\sigma :=m\alpha^{2}\nu^{-2}N_{0}\left(\gamma^{-1}{∥f_{1}∥}_{-1}+D_{c}^{-1}{∥f_{2}∥}_{-1}\right)+m\alpha^{2}\nu^{-1}\left(\gamma^{-2}N_{1}{∥f_{1}∥}_{-1}+D_{c}^{-2}N_{2}{∥f_{2}∥}_{-1}\right)<1, \end{array}$$

*then (u,p,T,C) is unique solution pair of *(5)*.*

**Proof.**  Suppose $\left(u_{1},p_{1},T_{1},C_{1}\right)$ is also a solution pair of (5) and $u_{1}\neq u,p_{1}\neq p,T_{1}\neq T,C_{1}\neq C,$ then

(11) $$\begin{array}{cc} a_{0}\left(u_{1},v\right)+c_{0}\left(u_{1},u_{1},v\right)+D_{a}^{-1}\left(u_{1},v\right)-d\left(p_{1},v\right)+d\left(q,u_{1}\right) & =(\beta_{T}T_{1}g+\beta_{C}C_{1}g,v),\;\\ a_{0}\left(u,v\right)+c_{0}\left(u,u,v\right)+D_{a}^{-1}\left(u,v\right)-d\left(p,v\right)+d\left(q,u\right) & =(\beta_{T}Tg+\beta_{C}Cg,v),\; \end{array}$$

for all (v,q)∈X×M.

Now, choosing $v=u-u_{1}$ and $q=p-p_{1},$ we obtain

$$\begin{array}{cc} \; & \;a_{0}\left(u-u_{1},u-u_{1}\right)+D_{a}^{-1}\left(u-u_{1},u-u_{1}\right)\\ \; & =-c_{0}\left(u-u_{1},u,u-u_{1}\right)+\left(\beta_{T}\left(T-T_{1}\right)g+\beta_{C}\left(C-C_{1}\right)g,u-u_{1}\right). \end{array}$$

Hence, applying (4), (9), (10), Theorem 1 and the uniqueness condition, we have

$$\begin{array}{cc} \nu {∥\nabla (u-u_{1})∥}_{0}^{2} & \leq \left(N_{0}{∥\nabla u∥}_{0}+m\alpha^{2}\left(\gamma^{-1}N_{1}{∥\nabla T∥}_{0}+D_{c}^{-1}N_{2}{∥\nabla C∥}_{0}\right)\right){∥\nabla (u-u_{1})∥}_{0}^{2}\\ & <\nu {∥\nabla (u-u_{1})∥}_{0}^{2}, \end{array}$$

a contradiction. Hence, $u_{1}=u,\;T_{1}=T,\;C_{1}=C$.  □

## 4. Several Iterative Methods Based on the Finite Element Discretization

In this section, we propose three iterative methods for the double-diffusive natural convection model. Then the stability and convergence of these iterative methods are considered. First, let 0<h<1 denote the mesh size which is a real positive parameter and $K_{h}=\left\{K:{⋃}_{K\subset \Omega }\bar{K}=\bar{\Omega }\right\}$ be a uniform partition of $\bar{\Omega }$ into non-overlapping triangles. Next, given a $K_{h}$, we consider the finite element spaces $X_{h},M_{h}$, $W_{h}$ and $Q_{h}$

$$\begin{array}{cc} & V_{h}=\left\{v_{h}\in V\cap C^{0}{\left(\bar{\Omega }\right)}^{2}:v_{h}{|}_{K}\in P_{2}{\left(K\right)}^{2},\;\forall K\in K_{h}\right\},\\ & M_{h}=\left\{q_{h}\in M\cap C^{0}\left(\bar{\Omega }\right):q_{h}{|}_{K}\in P_{1}\left(K\right),\;\;\;\forall K\in K_{h}\right\},\\ & W_{h}=\left\{\psi_{h}\in W\cap C^{0}\left(\bar{\Omega }\right):\psi_{h}{|}_{K}\in P_{2}\left(K\right),\;\;\;\forall K\in K_{h}\right\},\\ & Q_{h}=\left\{s_{h}\in Q\cap C^{0}\left(\bar{\Omega }\right):s_{h}{|}_{K}\in P_{2}\left(K\right),\;\;\;\;\;\forall K\in K_{h}\right\}, \end{array}$$

where $P_{i}\left(K\right)$ represents the space of the order polynomial on the set $K_{h}$, i=1,2. Please note that the Taylor-Hood element $X_{h}\times M_{h}$ satisfies the following discret inf-sup condition

$$\underset{v\in X_{h}}{\sup}\frac{\left|d(q,v)\right|}{{∥\nabla v∥}_{0}}\geq \beta {∥q∥}_{0},\;\forall q\in M_{h},$$

where the constant β>0 is independent of *h*.

With the above notations, the finite element scheme for the natural convection problem is defined as follows: find $(u_{h},p_{h},T_{h},C_{h})\in X\times M\times W\times Q$ such that

(12) $$\left\{\begin{array}{c} a_{0}\left(u_{h},v\right)+c_{0}\left(u_{h},u_{h},v\right)+D_{a}^{-1}\left(u_{h},v\right)-d\left(p_{h},v\right)+d\left(q,u_{h}\right)\\ \;=(\beta_{T}T_{h}g+\beta_{C}C_{h}g,v),\\ a_{1}\left(T_{h},\psi \right)+c_{1}\left(u_{h},T_{h},\psi \right)=\left(f_{1},\psi \right),\\ a_{2}\left(C_{h},s\right)+c_{2}\left(u_{h},C_{h},s\right)=\left(f_{2},s\right), \end{array}\right.$$

for all $\left(v,q,\psi ,s\right)\in X_{h}\times M_{h}\times W_{h}\times Q_{h}.$ The following stability and convergence results of the numerical solutions to (12) are showed.

**Theorem** **3.** ([7,8,26,27]) *Let $\left(u,p,T,C\right)\in \left(H^{3}{\left(\Omega \right)}^{2}\cap X\right)\times \left(H^{2}\left(\Omega \right)\cap M\right)\times \left(H^{3}\left(\Omega \right)\cap W\right)\times \left(H^{3}\left(\Omega \right)\cap Q\right)$. Under the assumption of Theorem 2, the numerical solution pair $\left(u_{h},p_{h},T_{h},C_{h}\right)$ to *(12)* satisfies*

$${∥\nabla T_{h}∥}_{0}\leq \gamma^{-1}{∥f_{1}∥}_{-1},\;\;\;{∥\nabla C_{h}∥}_{0}\leq D_{c}^{-1}{∥f_{2}∥}_{-1},$$

*and*

$${∥\nabla u_{h}∥}_{0}\leq \nu^{-1}m\alpha \left(\gamma^{-1}{∥f_{1}∥}_{-1}+D_{c}^{-1}{∥f_{2}∥}_{-1}\right).$$

*Moreover, the following error estimate holds*

$$\begin{array}{cc} \; & {∥\nabla (u-u_{h})∥}_{0}+{∥(p-p_{h})∥}_{0}+{∥\nabla (T-T_{h})∥}_{0}+{∥\nabla (C-C_{h})∥}_{0}\\ \leq & ch^{2}\left({∥u∥}_{3}+{∥p∥}_{2}+{∥T∥}_{3}+{∥C∥}_{3}\right), \end{array}$$

*where c is a positive constant depending on h*.

In the following part of this section, we propose and analyse three iterative methods.

**Iterative method I**. Find $\left(u_{h}^{n},p_{h}^{n},T_{h}^{n},C_{h}^{n}\right)\in X_{h}\times M_{h}\times W_{h}\times Q_{h}$ such that

(13) $$\left\{\begin{array}{c} a_{0}\left(u_{h}^{n},v\right)+c_{0}\left(u_{h}^{n-1},u_{h}^{n},v\right)+D_{a}^{-1}\left(u_{h}^{n},v\right)-d\left(p_{h}^{n},v\right)+d\left(q,u_{h}^{n}\right)\\ \;=(\beta_{T}T_{h}^{n}g+\beta_{C}C_{h}^{n}g,v),\\ a_{1}\left(T_{h}^{n},\psi \right)+c_{1}\left(u_{h}^{n-1},T_{h}^{n},\psi \right)=\left(f_{1},\psi \right),\\ a_{2}\left(C_{h}^{n},s\right)+c_{2}\left(u_{h}^{n-1},C_{h}^{n},s\right)=\left(f_{2},s\right), \end{array}\right.$$

for all $\left(v_{h},q,\psi ,s\right)\in X_{h}\times M_{h}\times W_{h}\times Q_{h}$.

**Iterative method II**. Find $\left(u_{h}^{n},p_{h}^{n},T_{h}^{n},C_{h}^{n}\right)\in X_{h}\times M_{h}\times W_{h}\times Q_{h}$ such that

(14) $$\left\{\begin{array}{c} a_{0}\left(u_{h}^{n},v\right)+c_{0}\left(u_{h}^{n-1},u_{h}^{n},v\right)+c_{0}\left(u_{h}^{n},u_{h}^{n-1},v\right)-c_{0}\left(u_{h}^{n-1},u_{h}^{n-1},v\right)+D_{a}^{-1}\left(u_{h}^{n},v\right)\\ \;-d\left(v,p_{h}^{n}\right)+d\left(u_{h}^{n},q\right)=\left(\beta_{T}T_{h}^{n}g+\beta_{C}C_{h}^{n}g,v\right),\\ a_{1}\left(T_{h}^{n},\psi \right)+c_{1}\left(u_{h}^{n-1},T_{h}^{n},\psi \right)+c_{1}\left(u_{h}^{n},T_{h}^{n-1},\psi \right)-c_{1}\left(u_{h}^{n-1},T_{h}^{n-1},\psi \right)=\left(f_{1},\psi \right),\\ a_{2}\left(C_{h}^{n},s\right)+c_{2}\left(u_{h}^{n-1},C_{h}^{n},s\right)+c_{2}\left(u_{h}^{n},C_{h}^{n-1},s\right)-c_{2}\left(u_{h}^{n-1},C_{h}^{n-1},s\right)=\left(f_{2},s\right), \end{array}\right.$$

for all $\left(v,q,\psi ,s\right)\in X_{h}\times M_{h}\times W_{h}\times Q_{h}.$

**Iterative method III**. Find $\left(u_{h}^{n},p_{h}^{n},T_{h}^{n},C_{h}^{n}\right)\in X_{h}\times M_{h}\times W_{h}\times Q_{h}$ such that

(15) $$\left\{\begin{array}{c} a_{0}\left(u_{h}^{n},v\right)+c_{0}\left(u_{h}^{n-1},u_{h}^{n-1},v\right)+D_{a}^{-1}\left(u_{h}^{n},v\right)-d\left(p_{h}^{n},v\right)+d\left(q,u_{h}^{n}\right)\\ \;=(\beta_{T}T_{h}^{n}g+\beta_{C}C_{h}^{n}g,v),\\ a_{1}\left(T_{h}^{n},\psi \right)+c_{1}\left(u_{h}^{n-1},T_{h}^{n-1},\psi \right)=\left(f_{1},\psi \right),\\ a_{2}\left(C_{h}^{n},s\right)+c_{2}\left(u_{h}^{n-1},C_{h}^{n-1},s\right)=\left(f_{2},s\right), \end{array}\right.$$

for all $\left(v,q,\psi ,s\right)\in X_{h}\times M_{h}\times W_{h}\times Q_{h}.$

For the above three iterative methods, the initial guess $\left(u_{h}^{0},p_{h}^{0},T_{h}^{0},C_{h}^{0}\right)\in X_{h}\times M_{h}\times W_{h}\times Q_{h}$ is defined by solving the following equations

(16) $$\left\{\begin{array}{c} a_{0}\left(u_{h}^{0},v\right)+D_{a}^{-1}\left(u_{h}^{0},v\right)-d\left(p_{h}^{0},v\right)+d\left(q,u_{h}^{0}\right)=\left(\beta_{T}T_{h}^{0}g+\beta_{C}C_{h}^{0}g,v\right),\\ a_{1}\left(T_{h}^{0},\psi \right)=\left(f_{1},\psi \right),\\ a_{2}\left(C_{h}^{0},s\right)=\left(f_{2},s\right), \end{array}\right.$$

for all $\left(v,q,\psi ,s\right)\in X_{h}\times M_{h}\times W_{h}\times Q_{h}.$

Now, we will establish the stability and iterative error estimates of the presented iterative methods for the double-diffusive natural convection model. For the sake of simplicity, let $\left(e^{n},\eta^{n},\xi^{n},\delta^{n}\right)=\left(u_{h}-u_{h}^{n},p_{h}-p_{h}^{n},T_{h}-T_{h}^{n},C_{h}-C_{h}^{n}\right).$

**Theorem** **4.** *Under the assumptions of Theorem 3, $\left(u_{h}^{n},p_{h}^{n},T_{h}^{n},C_{h}^{n}\right)$ defined by iterative method I satisfies*

(17) $$\begin{array}{cc} {∥\nabla u_{h}^{n}∥}_{0} & \leq \nu^{-1}m\alpha^{2}\left(\gamma^{-1}{∥f_{1}∥}_{-1}+D_{c}^{-1}{∥f_{2}∥}_{-1}\right),\\ {∥\nabla T_{h}^{n}∥}_{0} & \leq \gamma^{-1}{∥f_{1}∥}_{-1},\;{∥\nabla C_{h}^{n}∥}_{0}\leq D_{c}^{-1}{∥f_{2}∥}_{-1}, \end{array}$$

*for all n≥0. Furthermore, the following iterative error bounds hold*

(18) $$\begin{array}{cc} {∥\nabla e^{n}∥}_{0} & \leq \sigma^{n}\nu^{-1}m\alpha^{2}\left(\gamma^{-1}{∥f_{1}∥}_{-1}+D_{c}^{-1}{∥f_{2}∥}_{-1}\right),\\ {∥\nabla \eta^{n}∥}_{0} & \leq 4\beta^{-1}\sigma^{n}m\alpha^{2}\left(\gamma^{-1}{∥f_{1}∥}_{-1}+D_{c}^{-1}{∥f_{2}∥}_{-1}\right),\\ {∥\nabla \xi^{n}∥}_{0} & \leq \sigma^{n}\gamma^{-1}{∥f_{1}∥}_{-1},\;{∥\nabla \delta^{n}∥}_{0}\leq \sigma^{n}D_{c}^{-1}{∥f_{2}∥}_{-1}, \end{array}$$

*for all n≥0.*

**Proof.**  First, the induction method is used to consider the stability of iterative method I. Setting $\left(v,q,\psi ,s\right)=\left(u_{h}^{0},p_{h}^{0},T_{h}^{0},C_{h}^{0}\right)$ in (16) leads to

(19) $$\begin{array}{cc} {∥\nabla T_{h}^{0}∥}_{0} & \leq \gamma^{-1}{∥f_{1}∥}_{-1},\;{∥\nabla C_{h}^{0}∥}_{0}\leq D_{c}^{-1}{∥f_{2}∥}_{-1},\\ {∥\nabla u_{h}^{0}∥}_{0} & \leq \nu^{-1}m\alpha^{2}\left({∥\nabla T_{h}^{0}∥}_{0}+{∥\nabla C_{h}^{0}∥}_{0}\right)\\ \; & \leq \nu^{-1}m\alpha^{2}\left(\gamma^{-1}{∥f_{1}∥}_{-1}+D_{c}^{-1}{∥f_{2}∥}_{-1}\right). \end{array}$$

which shows that (17) holds for n=0.

Next, assuming that it holds for n=k, we prove that it is valid for n=k+1. Taking $\left(v,q,\psi ,s\right)=\left(u_{h}^{k+1},p_{h}^{k+1},T_{h}^{k+1},C_{h}^{k+1}\right)$ in (13) with n=k+1 and applying (2), (3) and (4) yield

$$\begin{array}{cc} {∥\nabla T_{h}^{k+1}∥}_{0} & \leq \gamma^{-1}{∥f_{1}∥}_{-1},\;{∥\nabla C_{h}^{k+1}∥}_{0}\leq D_{c}^{-1}{∥f_{2}∥}_{-1},\\ {∥\nabla u_{h}^{k+1}∥}_{0} & \leq \nu^{-1}m\alpha^{2}\left({∥\nabla T_{h}^{k+1}∥}_{0}+{∥\nabla C_{h}^{k+1}∥}_{0}\right). \end{array}$$

Hence, we finish the induction method.

Moreover, we consider the iterative error estimates of iterative method I. Making use of (12) and (13) yields the error equations

(20) $$\begin{array}{c} a_{0}\left(e^{n},v\right)+c_{0}\left(u_{h}^{n-1},e^{n},v\right)+c_{0}\left(e^{n-1},u_{h},v\right)+D_{a}^{-1}\left(e^{n},v\right)-d\left(\eta^{n},v\right)+d\left(q,e^{n}\right)\\ =(\beta_{T}\xi^{n}g+\beta_{C}\delta^{n}g,v),\\ a_{1}\left(\xi^{n},\psi \right)+c_{1}\left(u_{h}^{n-1},\xi^{n},\psi \right)+c_{1}\left(e^{n-1},T_{h},\psi \right)=0,\\ a_{2}\left(\delta^{n},s\right)+c_{2}\left(u_{h}^{n-1},\delta^{n},s\right)+c_{2}\left(e^{n-1},C_{h},s\right)=0. \end{array}$$

Setting $\psi =\xi^{n}$, $s=\delta^{n}$ in the second and the third equation of (20) and using (3), (17), and Theorem 3, we obtain

(21) $$\begin{array}{cc} {∥\nabla \xi^{n}∥}_{0} & \leq N_{1}\gamma^{-2}{∥f_{1}∥}_{-1}{∥\nabla e^{n-1}∥}_{0},\;\forall n\geq 1,\\ {∥\nabla \delta^{n}∥}_{0} & \leq N_{2}D_{c}^{-2}{∥f_{2}∥}_{-1}{∥\nabla e^{n-1}∥}_{0},\;\forall n\geq 1. \end{array}$$

Then, taking $\left(v,q\right)=\left(e^{n},\eta^{n}\right)$ in the first equation of (20) and using (2), (4), (17), (21) and Theorem 3, we arrive at

$$\begin{array}{cc} \nu {∥\nabla e^{n}∥}_{0}\leq & N_{0}{∥\nabla e^{n-1}∥}_{0}{∥\nabla u_{h}∥}_{0}+m\alpha^{2}\left({∥\nabla \xi^{n}∥}_{0}+{∥\nabla \delta^{n}∥}_{0}\right)\\ \leq & N_{0}{∥\nabla e^{n-1}∥}_{0}\nu^{-1}m\alpha^{2}\left(\gamma^{-1}{∥f_{1}∥}_{-1}+D_{c}^{-1}{∥f_{2}∥}_{-1}\right)\\ & +m\alpha^{2}\left(N_{1}\gamma^{-2}{∥f_{1}∥}_{-1}+N_{2}D_{c}^{-2}{∥f_{2}∥}_{-1}\right){∥\nabla e^{n-1}∥}_{0}. \end{array}$$

Hence, using uniqueness condition, we have

(22) $${∥\nabla e^{n}∥}_{0}\leq \sigma {∥\nabla e^{n-1}∥}_{0}\leq \sigma^{n}{∥\nabla e^{0}∥}_{0},\;\forall n\geq 1.$$

Furthermore, subtracting (16) from (12), we obtain

$$\begin{array}{cc} a_{0}\left(e^{0},v\right)+c_{0}\left(u_{h},u_{h},v\right)+D_{a}^{-1}\left(e^{0},v\right)-d\left(\eta^{0},v\right)+d\left(q,e^{0}\right) & =(\beta_{T}\xi^{0}g+\beta_{C}\delta^{0}g,v),\\ a_{1}\left(\xi^{0},\psi \right)+c_{1}\left(u_{h},T_{h},\psi \right) & =0,\\ a_{2}\left(\delta^{0},s\right)+c_{2}\left(u_{h},C_{h},s\right) & =0. \end{array}$$

Applying (4), the Theorem 2 and the Theorem 3, we obtain

(23) $$\begin{array}{cc} {∥\nabla \xi^{0}∥}_{0}\leq & N_{1}\gamma^{-2}\nu^{-1}m\alpha^{2}\left(\gamma^{-1}{∥f_{1}∥}_{-1}+D_{c}^{-1}{∥f_{2}∥}_{-1}\right){∥f_{1}∥}_{-1}\leq \gamma^{-1}{∥f_{1}∥}_{-1},\\ {∥\nabla \delta^{0}∥}_{0}\leq & N_{2}D_{c}^{-2}\nu^{-1}m\alpha^{2}\left(\gamma^{-1}{∥f_{1}∥}_{-1}+D_{c}^{-1}{∥f_{2}∥}_{-1}\right){∥f_{2}∥}_{-1}\leq D_{c}^{-1}{∥f_{2}∥}_{-1},\\ {∥\nabla e^{0}∥}_{0}\leq & N_{0}\nu^{-3}m^{2}\alpha^{4}{\left(\gamma^{-1}{∥f_{1}∥}_{-1}+D_{c}^{-1}{∥f_{2}∥}_{-1}\right)}^{2}\\ \; & +N_{1}\gamma^{-2}\nu^{-1}m^{2}\alpha^{4}\left(\gamma^{-1}{∥f_{1}∥}_{-1}+D_{c}^{-1}{∥f_{2}∥}_{-1}\right){∥f_{1}∥}_{-1}\\ \; & +N_{2}D_{c}^{-2}\nu^{-1}m^{2}\alpha^{4}\left(\gamma^{-1}{∥f_{1}∥}_{-1}+D_{c}^{-1}{∥f_{2}∥}_{-1}\right){∥f_{2}∥}_{-1},\\ \leq & \nu^{-1}m\alpha \left(\gamma^{-1}{∥f_{1}∥}_{-1}+D_{c}^{-1}{∥f_{2}∥}_{-1}\right), \end{array}$$

which combines with (21) and (22), we arrive at

$$\begin{array}{cc} {∥\nabla e^{n}∥}_{0} & \leq \sigma^{n}\nu^{-1}m\alpha^{2}\left(\gamma^{-1}{∥f_{1}∥}_{-1}+D_{c}^{-1}{∥f_{2}∥}_{-1}\right),\\ {∥\nabla \xi^{n}∥}_{0} & \leq N_{1}\gamma^{-2}{∥f_{1}∥}_{-1}\sigma^{n-1}\nu^{-1}m\alpha^{2}\left(\gamma^{-1}{∥f_{1}∥}_{-1}+D_{c}^{-1}{∥f_{2}∥}_{-1}\right)\\ & \leq \sigma^{n}\gamma^{-1}{∥f_{1}∥}_{-1},\\ {∥\nabla \delta^{n}∥}_{0} & \leq \;N_{2}D_{c}^{-2}{∥f_{2}∥}_{-1}\sigma^{n-1}\nu^{-1}m\alpha^{2}\left(\gamma^{-1}{∥f_{1}∥}_{-1}+D_{c}^{-1}{∥f_{2}∥}_{-1}\right),\\ & \leq \sigma^{n}D_{c}^{-1}{∥f_{2}∥}_{-1}, \end{array}$$

for all n≥0.

Finally, applying the discrete inf-sup condition, from the first equation of (20) with q=0, the error estimate of the pressure can be stated as follows.

$$\begin{array}{cc} {∥\eta^{n}∥}_{0}\leq & \beta^{-1}\left(\nu {∥\nabla e^{n}∥}_{0}+N_{0}{∥\nabla u_{h}^{n-1}∥}_{0}{∥\nabla e^{n}∥}_{0}+N_{0}{∥\nabla e^{n-1}∥}_{0}{∥\nabla u_{h}∥}_{0}\right)\\ & +\beta^{-1}\left(m\alpha^{2}\left({∥\nabla \xi^{n}∥}_{0}+{∥\nabla \delta^{n}∥}_{0}\right)\right)\\ \leq & \beta^{-1}(\sigma^{n}m\alpha^{2}\left(\gamma^{-1}{∥f_{1}∥}_{-1}+D_{c}^{-1}{∥f_{2}∥}_{-1}\right)\\ & +N_{0}\nu^{-2}m^{2}\alpha^{4}\left(\sigma^{n}+\sigma^{n-1}\right){\left(\gamma^{-1}{∥f_{1}∥}_{-1}+D_{c}^{-1}{∥f_{2}∥}_{-1}\right)}^{2}\\ & +m\alpha^{2}\sigma^{n}\left(\gamma^{-1}{∥f_{1}∥}_{-1}+D_{c}^{-1}{∥f_{2}∥}_{-1}\right))\\ \leq & 4\beta^{-1}\sigma^{n}m\alpha^{2}\left(\gamma^{-1}{∥f_{1}∥}_{-1}+D_{c}^{-1}{∥f_{2}∥}_{-1}\right), \end{array}$$

for all n≥0.  □

**Theorem** **5.** *Under the assumptions of Theorem *3*, suppose that the following condition (the strong uniqueness condition)*

(24) $$0<\sigma <\frac{1}{3},$$

*holds. Then $\left(u_{h}^{n},p_{h}^{n},T_{h}^{n},C_{h}^{n}\right)$ generated by iterative method II satisfies*

(25) $$\begin{array}{cc} {∥\nabla T_{h}^{n}∥}_{0} & \leq \frac{4}{3}\gamma^{-1}{∥f_{1}∥}_{-1},\;{∥\nabla C_{h}^{n}∥}_{0}\leq \frac{4}{3}D_{c}^{-1}{∥f_{2}∥}_{-1},\\ {∥\nabla u_{h}^{n}∥}_{0} & \leq \frac{4}{3}\nu^{-1}m\alpha^{2}\left(\gamma^{-1}{∥f_{1}∥}_{-1}+D_{c}^{-1}{∥f_{2}∥}_{-1}\right), \end{array}$$

*for all n≥0. Furthermore, the following iterative error bounds hold*

(26) $$\begin{array}{cc} {∥\nabla e^{n}∥}_{0} & \leq {\left(\frac{9}{5}\sigma \right)}^{2^{n}-1}\nu^{-1}m\alpha^{2}\left(\gamma^{-1}{∥f_{1}∥}_{-1}+D_{c}^{-1}{∥f_{2}∥}_{-1}\right),\\ {∥\nabla \eta^{n}∥}_{0} & \leq \frac{119}{45}\beta^{-1}{\left(\frac{9}{5}\sigma \right)}^{2^{n}-1}m\alpha^{2}\left(\gamma^{-1}{∥f_{1}∥}_{-1}+D_{c}^{-1}{∥f_{2}∥}_{-1}\right),\\ {∥\nabla \xi^{n}∥}_{0} & \leq {\left(\frac{9}{5}\sigma \right)}^{2^{n}-1}\gamma^{-1}{∥f_{1}∥}_{-1},\;{∥\nabla \delta^{n}∥}_{0}\leq {\left(\frac{9}{5}\sigma \right)}^{2^{n}-1}D_{c}^{-1}{∥f_{2}∥}_{-1}, \end{array}$$

*for all n≥0.*

**Proof.**  Combining with (19) and (23), it is found that (25) and (26) hold for n=0. Supposing that (25) and (26) hold for n=k, we shall prove that they are valid for n=k+1.

Subtracting (14) from (12), we obtain the error equations

(27) $$\begin{array}{cc} a_{0}(e^{n} & ,v)+c_{0}\left(u_{h}^{n-1},e^{n},v\right)+c_{0}\left(e^{n},u_{h}^{n-1},v\right)+c_{0}\left(e^{n-1},e^{n-1},v\right)+D_{a}^{-1}\left(e^{n},v\right)\\ \; & -d\left(v,\eta^{n}\right)+d\left(e^{n},q\right)=\left(\beta_{T}\xi^{n}g+\beta_{C}\delta^{n}g,v\right),\\ a_{1}(\xi^{n} & ,\psi )+c_{1}\left(u_{h}^{n-1},\xi^{n},\psi \right)+c_{1}\left(e^{n},T_{h}^{n-1},\psi \right)+c_{1}\left(e^{n-1},\xi^{n-1},\psi \right)=0,\\ a_{2}(\sigma & {}^{n},s)+c_{2}\left(u_{h}^{n-1},\delta^{n},s\right)+c_{2}\left(e^{n},C_{h}^{n-1},s\right)+c_{2}\left(e^{n-1},\delta^{n-1},s\right)=0. \end{array}$$

Setting $\left(v,q,\psi ,s\right)=\left(e_{h}^{n},\eta_{h}^{n},\xi_{h}^{n},\delta_{h}^{n}\right)$ in (27) with n=k+1 and applying (2), (3), (4) and the assumptions on n=k, we have

(28) $$\begin{array}{cc} {∥\nabla \xi^{k+1}∥}_{0} & \leq N_{1}\gamma^{-1}{∥\nabla e^{k+1}∥}_{0}{∥\nabla T_{h}^{k}∥}_{0}+N_{1}\gamma^{-1}{∥\nabla e^{k}∥}_{0}{∥\nabla \xi^{k}∥}_{0}\\ \; & \leq \frac{4}{3}N_{1}\gamma^{-2}{∥f_{1}∥}_{-1}{∥\nabla e^{k+1}∥}_{0}+N_{1}\gamma^{-1}{∥\nabla e^{k}∥}_{0}{∥\nabla \xi^{k}∥}_{0},\\ {∥\nabla \delta^{k+1}∥}_{0} & \leq N_{2}D_{c}^{-1}{∥\nabla e^{k+1}∥}_{0}{∥\nabla C_{h}^{k}∥}_{0}+N_{2}D_{c}^{-1}{∥\nabla e^{k}∥}_{0}{∥\nabla \delta^{k}∥}_{0}\\ \; & \leq \frac{4}{3}N_{2}D_{c}^{-2}{∥f_{2}∥}_{-1}{∥\nabla e^{k+1}∥}_{0}+N_{2}D_{c}^{-1}{∥\nabla e^{k}∥}_{0}{∥\nabla \delta^{k}∥}_{0}, \end{array}$$

and

(29) $$\begin{array}{cc} \nu {∥\nabla e^{k+1}∥}_{0}\leq & N_{0}{∥\nabla u_{h}^{k}∥}_{0}{∥\nabla e^{k+1}∥}_{0}+N_{0}{∥\nabla e^{k}∥}_{0}^{2}+m\alpha^{2}\left({∥\nabla \xi^{k+1}∥}_{0}+{∥\nabla \delta^{k+1}∥}_{0}\right)\\ \leq & \frac{4}{3}N_{0}\nu^{-1}m\alpha^{2}\left(\gamma^{-1}{∥f_{1}∥}_{-1}+D_{c}^{-1}{∥f_{2}∥}_{-1}\right){∥\nabla e^{k+1}∥}_{0}+N{∥\nabla e^{k}∥}_{0}^{2}\\ \; & +m\alpha^{2}\left(\frac{4}{3}N_{1}\gamma^{-2}{∥f_{1}∥}_{-1}{∥\nabla e^{k+1}∥}_{0}+N_{1}\gamma^{-1}{∥\nabla e^{k}∥}_{0}{∥\nabla \xi^{k}∥}_{0}\right)\\ \; & +m\alpha^{2}\left(\frac{4}{3}N_{2}D_{c}^{-2}{∥f_{2}∥}_{-1}{∥\nabla e^{k+1}∥}_{0}+N_{2}D_{c}^{-1}{∥\nabla e^{k}∥}_{0}{∥\nabla \delta^{k}∥}_{0}\right). \end{array}$$

Moreover, imply the strong uniqueness condition (24) on (29), we obtain

(30) $$\begin{array}{cc} {∥\nabla e^{k+1}∥}_{0}\leq & \frac{9}{5}N_{0}\nu^{-1}{\left(\frac{9}{5}\sigma \right)}^{2^{k+1}-2}\nu^{-2}m^{2}\alpha^{4}{\left(\gamma^{-1}{∥f_{1}∥}_{-1}+D_{c}^{-1}{∥f_{2}∥}_{-1}\right)}^{2}\\ \; & +\frac{9}{5}\nu^{-1}m\alpha^{2}N_{1}{\left(\frac{9}{5}\sigma \right)}^{2^{k+1}-2}\nu^{-1}m\alpha^{2}\gamma^{-2}\left(\gamma^{-1}{∥f_{1}∥}_{-1}+D_{c}^{-1}{∥f_{2}∥}_{-1}\right){∥f_{1}∥}_{-1}\\ \; & +\frac{9}{5}\nu^{-1}m\alpha^{2}N_{2}{\left(\frac{9}{5}\sigma \right)}^{2^{k+1}-2}\nu^{-1}m\alpha^{2}D_{c}^{-2}\left(\gamma^{-1}{∥f_{1}∥}_{-1}+D_{c}^{-1}{∥f_{2}∥}_{-1}\right){∥f_{2}∥}_{-1}\\ \leq & {\left(\frac{9}{5}\sigma \right)}^{2^{k+1}-1}\nu^{-1}m\alpha^{2}\left(\gamma^{-1}{∥f_{1}∥}_{-1}+D_{c}^{-1}{∥f_{2}∥}_{-1}\right). \end{array}$$

Hence, making use of (30), we rewrite (28) as

(31) $$\begin{array}{cc} {∥\nabla \xi^{k+1}∥}_{0}\leq & \frac{4}{3}N_{1}\gamma^{-2}{∥f_{1}∥}_{-1}{\left(\frac{9}{5}\sigma \right)}^{2^{k+1}-1}\nu^{-1}m\alpha \left(\gamma^{-1}{∥f_{1}∥}_{-1}+D_{c}^{-1}{∥f_{2}∥}_{-1}\right)\\ \; & +N_{1}\gamma^{-1}{\left(\frac{9}{5}\sigma \right)}^{2^{k+1}-2}\nu^{-1}m\alpha \left(\gamma^{-1}{∥f_{1}∥}_{-1}+D_{c}^{-1}{∥f_{2}∥}_{-1}\right)\gamma^{-1}{∥f_{1}∥}_{-1}\\ \leq & {\left(\frac{9}{5}\sigma \right)}^{2^{k+1}-1}\gamma^{-1}{∥f_{1}∥}_{-1}.\\ {∥\nabla \delta^{k+1}∥}_{0}\leq & \frac{4}{3}N_{2}D_{c}^{-2}{∥f_{2}∥}_{-1}{\left(\frac{9}{5}\sigma \right)}^{2^{k+1}-1}\nu^{-1}m\alpha \left(\gamma^{-1}{∥f_{1}∥}_{-1}+D_{c}^{-1}{∥f_{2}∥}_{-1}\right)\\ \; & +N_{2}D_{c}^{-1}{\left(\frac{9}{5}\sigma \right)}^{2^{k+1}-2}\nu^{-1}m\alpha \left(\gamma^{-1}{∥f_{1}∥}_{-1}+D_{c}^{-1}{∥f_{2}∥}_{-1}\right)D_{c}^{-1}{∥f_{2}∥}_{-1}\\ \leq & {\left(\frac{9}{5}\sigma \right)}^{2^{k+1}-1}D_{c}^{-1}{∥f_{2}∥}_{-1}. \end{array}$$

Combining the first equation of (27) with n=k+1 and q=0 and the discrete inf-sup condition, we have

(32) $$\begin{array}{cc} {∥\eta^{k+1}∥}_{0}\leq & \beta^{-1}\left(\nu {∥\nabla e^{k+1}∥}_{0}+N_{0}{∥\nabla e^{k+1}∥}_{0}{∥\nabla u_{h}^{k}∥}_{0}+N_{0}{∥\nabla e^{k}∥}_{0}^{2}\right)\\ \; & +\beta^{-1}\left(m\alpha^{2}\left({∥\nabla \xi^{k+1}∥}_{0}+{∥\nabla \delta^{k+1}∥}_{0}\right)\right)\\ \leq & \beta^{-1}(\nu {\left(\frac{9}{5}\sigma \right)}^{2^{k+1}-1}\nu^{-1}m\alpha^{2}\left(\gamma^{-1}{∥f_{1}∥}_{-1}+D_{c}^{-1}{∥f_{2}∥}_{-1}\right)\\ \; & +N_{0}{\left(\frac{9}{5}\sigma \right)}^{2^{k+1}-1}\nu^{-1}m\alpha^{2}\left(\gamma^{-1}{∥f_{1}∥}_{-1}+D_{c}^{-1}{∥f_{2}∥}_{-1}\right)\\ & \times \frac{4}{3}\nu^{-1}m\alpha^{2}\left(\gamma^{-1}{∥f_{1}∥}_{-1}+D_{c}^{-1}{∥f_{2}∥}_{-1}\right)\\ \; & +N_{0}{\left(\frac{9}{5}\sigma \right)}^{2^{k+1}-2}\nu^{-2}m^{2}\alpha^{4}{\left(\gamma^{-1}{∥f_{1}∥}_{-1}+D_{c}^{-1}{∥f_{2}∥}_{-1}\right)}^{2}\\ \; & +m\alpha^{2}\left({\left(\frac{9}{5}\sigma \right)}^{2^{k+1}-1}\gamma^{-1}{∥f_{1}∥}_{-1}+{\left(\frac{9}{5}\sigma \right)}^{2^{k+1}-1}D_{c}^{-1}{∥f_{2}∥}_{-1}\right))\\ \leq & \frac{119}{45}\beta^{-1}{\left(\frac{9}{5}\sigma \right)}^{2^{k+1}-1}m\alpha^{2}\left(\gamma^{-1}{∥f_{1}∥}_{-1}+D_{c}^{-1}{∥f_{2}∥}_{-1}\right). \end{array}$$

Furthermore, subtracting (16) from (14) with n=1 that

(33) $$\begin{array}{cc} a_{0}(u_{h}^{1} & -u_{h}^{0},v)+c_{0}\left(u_{h}^{0},u_{h}^{1}-u_{h}^{0},v\right)+c_{0}\left(u_{h}^{1},u_{h}^{0},v\right)+D_{a}^{-1}\left(u_{h}^{1}-u_{h}^{0},v\right)-d\left(v,p_{h}^{1}-p_{h}^{0}\right)\\ \; & +d\left(u_{h}^{1}-u_{h}^{0},q\right)=\left(\beta_{T}\left(T_{h}^{1}-T_{h}^{0}\right)g+\beta_{C}\left(C_{h}^{1}-C_{h}^{0}\right)g,v\right),\\ a_{1}(T_{h}^{1} & -T_{h}^{0},\psi )+c_{1}\left(u_{h}^{0},T_{h}^{1}-T_{h}^{0},\psi \right)+c_{1}\left(u_{h}^{1},T_{h}^{0},\psi \right)=0,\\ a_{2}(C & {}_{h}^{1}-C_{h}^{0},s)+c_{2}\left(u_{h}^{0},C_{h}^{1}-C_{h}^{0},s\right)+c_{2}\left(u_{h}^{1},C_{h}^{0},s\right)=0. \end{array}$$

Then, taking $\psi =T_{h}^{1}-T_{h}^{0}$ in the second equation of (33), we observe that

$${∥\nabla (T_{h}^{1}-T_{h}^{0})∥}_{0}\leq N_{1}\gamma^{-1}{∥\nabla u_{h}^{1}∥}_{0}{∥\nabla T_{h}^{0}∥}_{0},$$

and

$${∥\nabla (C_{h}^{1}-C_{h}^{0})∥}_{0}\leq N_{2}D_{c}^{-1}{∥\nabla u_{h}^{1}∥}_{0}{∥\nabla C_{h}^{0}∥}_{0}.$$

Moreover, setting $v=u_{h}^{1}-u_{h}^{0}$ in the first equation of (33), we obtain

(34) $$\begin{array}{cc} {∥\nabla (u_{h}^{1}-u_{h}^{0})∥}_{0}\leq & \nu^{-1}N_{0}{∥\nabla u_{h}^{1}∥}_{0}{∥\nabla u_{h}^{0}∥}_{0}\\ \; & +\nu^{-1}m\alpha^{2}\left({∥\nabla (T_{h}^{1}-T_{h}^{0})∥}_{0}+{∥\nabla (C_{h}^{1}-C_{h}^{0})∥}_{0}\right)\\ \leq & N_{0}\nu^{-2}m\alpha^{2}\left(\gamma^{-1}{∥f_{1}∥}_{-1}+D_{c}^{-1}{∥f_{2}∥}_{-1}\right){∥\nabla u_{h}^{1}∥}_{0}\\ \; & +\nu^{-1}m\alpha^{2}\left(N_{1}\gamma^{-2}{∥f_{1}∥}_{-1}+N_{2}D_{c}^{-2}{∥f_{2}∥}_{-1}\right){∥\nabla u_{h}^{1}∥}_{0}\\ \leq & \sigma {∥\nabla u_{h}^{1}∥}_{0}. \end{array}$$

Combining (14) with n=1 and using (34), we obtain

$$\begin{array}{cc} {∥\nabla T_{h}^{1}∥}_{0}\leq & \gamma^{-1}N_{1}{∥\nabla (u_{h}^{1}-u_{h}^{0})∥}_{0}{∥\nabla T_{h}^{0}∥}_{0}+\gamma^{-1}{∥f_{1}∥}_{-1}\\ \leq & \gamma^{-2}N_{1}\sigma {∥f_{1}∥}_{-1}{∥\nabla u_{h}^{1}∥}_{0}+\gamma^{-1}{∥f_{1}∥}_{-1},\\ {∥\nabla C_{h}^{1}∥}_{0}\leq & D_{c}^{-1}N_{2}{∥\nabla (u_{h}^{1}-u_{h}^{0})∥}_{0}{∥\nabla C_{h}^{0}∥}_{0}+D_{c}^{-1}{∥f_{2}∥}_{-1}\\ \leq & D_{c}^{-2}N_{2}\sigma {∥f_{2}∥}_{-1}{∥\nabla u_{h}^{1}∥}_{0}+D_{c}^{-1}{∥f_{2}∥}_{-1},\\ {∥\nabla u_{h}^{1}∥}_{0}\leq & \nu^{-1}N_{0}{∥\nabla (u_{h}^{1}-u_{h}^{0})∥}_{0}{∥\nabla u_{h}^{0}∥}_{0}+\nu^{-1}m\alpha^{2}\left({∥\nabla T_{h}^{1}∥}_{0}+{∥\nabla C_{h}^{1}∥}_{0}\right)\\ \leq & \nu^{-2}N_{0}\sigma {∥\nabla u_{h}^{1}∥}_{0}m\alpha^{2}\left(\gamma^{-1}{∥f_{1}∥}_{-1}+D_{c}^{-1}{∥f_{2}∥}_{-1}\right)\\ & +\nu^{-1}m\alpha^{2}(\gamma^{-2}N_{1}\sigma {∥f_{1}∥}_{-1}{∥\nabla u_{h}^{1}∥}_{0}+\gamma^{-1}{∥f_{1}∥}_{-1}\\ & +D_{c}^{-2}N_{2}\sigma {∥f_{2}∥}_{-1}{∥\nabla u_{h}^{1}∥}_{0}+D_{c}^{-1}{∥f_{2}∥}_{-1})\\ \leq & \sigma^{2}{∥\nabla u_{h}^{1}∥}_{0}+\nu^{-1}m\alpha^{2}\left(\gamma^{-1}{∥f_{1}∥}_{-1}+D_{c}^{-1}{∥f_{2}∥}_{-1}\right). \end{array}$$

In view of the strong uniqueness condition (24), we arrive at

$$\begin{array}{cc} {∥\nabla u_{h}^{1}∥}_{0} & \leq \frac{9}{8}\nu^{-1}m\alpha^{2}\left(\gamma^{-1}{∥f_{1}∥}_{-1}+D_{c}^{-1}{∥f_{2}∥}_{-1}\right),\\ {∥\nabla T_{h}^{1}∥}_{0} & \leq \frac{9}{8}\gamma^{-1}{∥f_{1}∥}_{-1},\;{∥\nabla C_{h}^{1}∥}_{0}\leq \frac{9}{8}D_{c}^{-1}{∥f_{2}∥}_{-1}. \end{array}$$

Next, taking $\left(v,q,\psi ,s\right)=\left(u_{h}^{n},p_{h}^{n},T_{h}^{n},C_{h}^{n}\right)$ in (14) with n≥2, and using (2), (3) and (26), we obtain

$$\begin{array}{cc} & {∥\nabla T_{h}^{n}∥}_{0}\\ \leq & \gamma^{-1}c_{1}\left(u_{h}^{n}-u_{h}^{n-1},T_{h}^{n-1}-T_{h}^{n},\psi \right)+\gamma^{-1}{∥f_{1}∥}_{-1}\\ \leq & \gamma^{-1}N_{1}{∥\nabla (e^{n-1}-e^{n})∥}_{0}{∥\nabla (\xi^{n-1}-\xi^{n})∥}_{0}+\gamma^{-1}{∥f_{1}∥}_{-1}\\ \leq & \gamma^{-1}N_{1}{\left({\left(\frac{9}{5}\sigma \right)}^{3}+\frac{9}{5}\sigma \right)}^{2}\nu^{-1}m\alpha^{2}\left(\gamma^{-1}{∥f_{1}∥}_{-1}+D_{c}^{-1}{∥f_{2}∥}_{-1}\right)\gamma^{-1}{∥f_{1}∥}_{-1}+\gamma^{-1}{∥f_{1}∥}_{-1}\\ \leq & \gamma^{-1}N_{1}{\left({\left(\frac{3}{5}\right)}^{3}+\frac{3}{5}\right)}^{2}\nu^{-1}m\alpha^{2}\left(\gamma^{-1}{∥f_{1}∥}_{-1}+D_{c}^{-1}{∥f_{2}∥}_{-1}\right)\gamma^{-1}{∥f_{1}∥}_{-1}+\gamma^{-1}{∥f_{1}∥}_{-1}\\ \leq & \frac{4}{3}\gamma^{-1}{∥f_{1}∥}_{-1}. \end{array}$$

Similarly, we obtain

$${∥\nabla C_{h}^{n}∥}_{0}\leq \frac{4}{3}D_{c}^{-1}{∥f_{2}∥}_{-1}.$$

Finally, it has

$$\begin{array}{cc} \; & {∥\nabla u_{h}^{n}∥}_{0}\\ \leq & \nu^{-1}N_{0}{∥\nabla (e^{n-1}-e^{n})∥}_{0}^{2}+\nu^{-1}m\alpha^{2}\left({∥\nabla T_{h}^{n}∥}_{0}+{∥\nabla C_{h}^{n}∥}_{0}\right)\\ \leq & \nu^{-1}N_{0}{∥\nabla (e^{n-1}-e^{n})∥}_{0}^{2}+\nu^{-1}m\alpha^{2}\left(\gamma^{-1}N_{1}{∥\nabla (e^{n-1}-e^{n})∥}_{0}{∥\nabla (\xi^{n-1}-\xi^{n})∥}_{0}+\gamma^{-1}{∥f_{1}∥}_{-1}\right)\\ \; & +\nu^{-1}m\alpha^{2}\left(D_{c}^{-1}N_{2}{∥\nabla (e^{n-1}-e^{n})∥}_{0}{∥\nabla (\delta^{n-1}-\delta^{n})∥}_{0}+D_{c}^{-1}{∥f_{2}∥}_{-1}\right)\\ \leq & \nu^{-1}N_{0}{\left({\left(\frac{3}{5}\right)}^{3}+\frac{3}{5}\right)}^{2}\nu^{-2}m^{2}\alpha^{4}{\left(\gamma^{-1}{∥f_{1}∥}_{-1}+D_{c}^{-1}{∥f_{2})∥}_{-1}\right)}^{2}\\ \; & +\nu^{-1}m\alpha^{2}\left(\gamma^{-1}N_{1}{\left({\left(\frac{3}{5}\right)}^{3}+\frac{3}{5}\right)}^{2}\nu^{-1}m\alpha^{2}\left(\gamma^{-1}{∥f_{1}∥}_{-1}+D_{c}^{-1}{∥f_{2}∥}_{-1}\right)\gamma^{-1}{∥f_{1}∥}_{-1}+\gamma^{-1}{∥f_{1}∥}_{-1}\right)\\ \; & +\nu^{-1}m\alpha^{2}\left(D_{c}^{-1}N_{2}{\left({\left(\frac{3}{5}\right)}^{3}+\frac{3}{5}\right)}^{2}\nu^{-1}m\alpha^{2}\left(\gamma^{-1}{∥f_{1}∥}_{-1}+D_{c}^{-1}{∥f_{2}∥}_{-1}\right)D_{c}^{-1}{∥f_{2}∥}_{-1}+D_{c}^{-1}{∥f_{2}∥}_{-1}\right)\\ \leq & \frac{4}{3}\nu^{-1}m\alpha^{2}\left(\gamma^{-1}{∥f_{1}∥}_{-1}+D_{c}^{-1}{∥f_{2}∥}_{-1}\right). \end{array}$$

The proof is completed. □

**Theorem** **6.** *Under the assumptions of Theorem *3*, suppose that the following condition (the stronger uniqueness condition),*

(35) $$0<\sigma <\frac{1}{4},$$

*holds. Then $\left(u_{h}^{n},p_{h}^{n},T_{h}^{n},C_{h}^{n}\right)$ defined by the* iterative method III *satisfies*

(36) $$\begin{array}{cc} {∥\nabla u_{h}^{n}∥}_{0} & \leq 2\nu^{-1}m\alpha^{2}\left(\gamma^{-1}{∥f_{1}∥}_{-1}+D_{c}^{-1}{∥f_{2}∥}_{-1}\right),\\ {∥\nabla T_{h}^{n}∥}_{0} & \leq 2\gamma^{-1}{∥f_{1}∥}_{-1},\;{∥\nabla C_{h}^{n}∥}_{0}\leq 2D_{c}^{-1}{∥f_{2}∥}_{-1}, \end{array}$$

*for all n≥0. Furthermore, the following iterative error bounds hold*

(37) $$\begin{array}{cc} \; & {∥\nabla e^{n}∥}_{0}\leq {\left(3\sigma \right)}^{n}\nu^{-1}m\alpha^{2}\left(\gamma^{-1}{∥f_{1}∥}_{-1}+D_{c}^{-1}{∥f_{2}∥}_{-1}\right),\\ \; & {∥\nabla \eta^{n}∥}_{0}\leq 5\beta^{-1}{\left(3\sigma \right)}^{n}m\alpha^{2}\left(\gamma^{-1}{∥f_{1}∥}_{-1}+D_{c}^{-1}{∥f_{2}∥}_{-1}\right),\\ \; & {∥\nabla \xi^{n}∥}_{0}\leq {\left(3\sigma \right)}^{n}\gamma^{-1}{∥f_{1}∥}_{-1},\;{∥\nabla \delta^{n}∥}_{0}\leq {\left(3\sigma \right)}^{n}D_{c}^{-1}{∥f_{2}∥}_{-1}, \end{array}$$

*for all n≥0.*

**Proof.**  From (19) and (23), it is obvious that (36) and (37) hold for n=0. Supposing that (36) and (37) hold for n=k, we shall prove that they are valid for n=k+1.

Setting $\left(v,q,\psi ,s\right)=\left(u_{h}^{n},p_{h}^{n},T_{h}^{n},C_{h}^{n}\right)$ in (15) with n=k+1 and using (2), (3), (4) and (36), we obtain that

$$\begin{array}{cc} {∥\nabla T_{h}^{k+1}∥}_{0}\leq & \gamma^{-1}N_{1}{∥\nabla u_{h}^{k}∥}_{0}{∥\nabla T_{h}^{k}∥}_{0}+\gamma^{-1}{∥f_{1}∥}_{-1}\\ \leq & \gamma^{-1}N_{1}2\nu^{-1}m\alpha^{2}\left(\gamma^{-1}{∥f_{1}∥}_{-1}+D_{c}^{-1}{∥f_{2}∥}_{-1}\right)2\gamma^{-1}{∥f_{1}∥}_{-1}+\gamma^{-1}{∥f_{1}∥}_{-1}\\ \leq & 2\gamma^{-1}{∥f_{1}∥}_{-1},\\ {∥\nabla C_{h}^{k+1}∥}_{0}\leq & D_{c}^{-1}N_{2}{∥\nabla u_{h}^{k}∥}_{0}{∥\nabla C_{h}^{k}∥}_{0}+D_{c}^{-1}{∥f_{1}∥}_{-1}\\ \leq & D_{c}^{-1}N_{2}2\nu^{-1}m\alpha^{2}\left(\gamma^{-1}{∥f_{1}∥}_{-1}+D_{c}^{-1}{∥f_{2}∥}_{-1}\right)2D_{c}^{-1}{∥f_{2}∥}_{-1}+D_{c}^{-1}{∥f_{2}∥}_{-1}\\ \leq & 2D_{c}^{-1}{∥f_{2}∥}_{-1},\\ {∥\nabla u_{h}^{k+1}∥}_{0}\leq & \nu^{-1}N_{0}{∥\nabla u_{h}^{k}∥}_{0}^{2}+\nu^{-1}m\alpha^{2}\left({∥\nabla T_{h}^{k+1}∥}_{0}+{∥\nabla C_{h}^{k+1}∥}_{0}\right)\\ \leq & \nu^{-1}N_{0}{∥\nabla u_{h}^{k}∥}_{0}^{2}+\nu^{-1}m\alpha^{2}\left(\gamma^{-1}N_{1}{∥\nabla u_{h}^{m}∥}_{0}{∥\nabla T_{h}^{k}∥}_{0}+\gamma^{-1}{∥f_{1}∥}_{-1}\right)\\ & +\nu^{-1}m\alpha^{2}\left(D_{c}^{-1}N_{2}{∥\nabla u_{h}^{k}∥}_{0}{∥\nabla C_{h}^{k}∥}_{0}+D_{c}^{-1}{∥f_{1}∥}_{-1}\right)\\ \leq & \nu^{-1}N_{0}4\nu^{-2}m^{2}\alpha^{4}{\left(\gamma^{-1}{∥f_{1}∥}_{-1}+D_{c}^{-1}{∥f_{2}∥}_{-1}\right)}^{2}\\ & +4\nu^{-2}\gamma^{-2}m^{2}\alpha^{4}N_{1}\left(\gamma^{-1}{∥f_{1}∥}_{-1}+D_{c}^{-1}{∥f_{2}∥}_{-1}\right){∥f_{1}∥}_{-1}\\ & +\nu^{-1}m\alpha^{2}\gamma^{-1}{∥f_{1}∥}_{-1}+\nu^{-1}m\alpha^{2}D_{c}^{-1}{∥f_{2}∥}_{-1}\\ & +4\nu^{-2}D_{c}^{-2}m^{2}\alpha^{4}N_{2}\left(\gamma^{-1}{∥f_{1}∥}_{-1}+D_{c}^{-1}{∥f_{2}∥}_{-1}\right){∥f_{2}∥}_{-1}\\ \leq & 2\nu^{-1}m\alpha^{2}\left(\gamma^{-1}{∥f_{1}∥}_{-1}+D_{c}^{-1}{∥f_{2}∥}_{-1}\right). \end{array}$$

Hence, (36) is valid for n=k+1. Consequently, subtracting (15) from (12) yields

(38) $$\begin{array}{cc} a_{0}(e^{n} & ,v)+c_{0}\left(u_{h}^{n-1},e^{n-1},v\right)+c_{0}\left(e^{n-1},u_{h},v\right)+D_{a}^{-1}\left(e^{n},v\right)-d\left(\eta^{n},v\right)+d\left(q,e^{n}\right)\\ \; & =(\beta_{T}\xi^{n}g+\beta_{C}\delta^{n}g,v),\\ a_{1}(\xi^{n} & ,\psi )+c_{1}\left(u_{h}^{n-1},\xi^{n-1},\psi \right)+c_{1}\left(e^{n-1},T_{h},\psi \right)=0,\\ a_{2}(\delta & {}^{n},s)+c_{2}\left(u_{h}^{n-1},\delta^{n-1},s\right)+c_{2}\left(e^{n-1},C_{h},s\right)=0. \end{array}$$

Now, choosing $\psi =\xi^{n}$, in the second equation of (38) and using (3), (36), (37) and Theorem 3, we can deduce that

(39) $$\begin{array}{cc} \; & {∥\nabla \xi^{n}∥}_{0}\\ \leq & 2N_{1}\gamma^{-1}\nu^{-1}m\alpha^{2}\left(\gamma^{-1}{∥f_{1}∥}_{-1}+D_{c}^{-1}{∥f_{2}∥}_{-1}\right){∥\nabla \xi^{n-1}∥}_{0}+N_{1}\gamma^{-2}{∥f_{1}∥}_{-1}{∥\nabla e^{n-1}∥}_{0}\\ \leq & 2N_{1}\gamma^{-1}\nu^{-1}m\alpha^{2}\left(\gamma^{-1}{∥f_{1}∥}_{-1}+D_{c}^{-1}{∥f_{2}∥}_{-1}\right){\left(3\sigma \right)}^{n-1}\gamma^{-1}{∥f_{1}∥}_{-1}\\ \; & +N_{1}\gamma^{-2}{∥f_{1}∥}_{-1}{\left(3\sigma \right)}^{n-1}\nu^{-1}m\alpha^{2}\left(\gamma^{-1}{∥f_{1}∥}_{-1}+D_{c}^{-1}{∥f_{2}∥}_{-1}\right)\\ \leq & {\left(3\sigma \right)}^{n}\gamma^{-1}{∥f_{1}∥}_{-1},\;\;\;\forall n\geq 1. \end{array}$$

Similarly, one has

(40) $$\begin{array}{cc} \; & {∥\nabla \delta^{n}∥}_{0}\\ \leq & 2N_{2}D_{c}^{-1}\nu^{-1}m\alpha^{2}\left(\gamma^{-1}{∥f_{1}∥}_{-1}+D_{c}^{-1}{∥f_{2}∥}_{-1}\right){∥\nabla \delta^{n-1}∥}_{0}+N_{2}D_{c}^{-2}{∥f_{2}∥}_{-1}{∥\nabla e^{n-1}∥}_{0}\\ \leq & 2N_{2}D_{c}^{-1}\nu^{-1}m\alpha^{2}\left(\gamma^{-1}{∥f_{1}∥}_{-1}+D_{c}^{-1}{∥f_{2}∥}_{-1}\right){\left(3\sigma \right)}^{n-1}D_{c}^{-1}{∥f_{2}∥}_{-1}\\ \; & +N_{2}D_{c}^{-2}{∥f_{2}∥}_{-1}{\left(3\sigma \right)}^{n-1}\nu^{-1}m\alpha^{2}\left(\gamma^{-1}{∥f_{1}∥}_{-1}+D_{c}^{-1}{∥f_{2}∥}_{-1}\right)\\ \leq & {\left(3\sigma \right)}^{n}D_{c}^{-1}{∥f_{2}∥}_{-1},\;\;\;\forall n\geq 1. \end{array}$$

Moreover, taking $\left(v,q\right)=\left(e^{n},\eta^{n}\right)$ in the first equation of (38) and using (2), (4), (36), (37) and the Theorem 3, we find that

(41) $$\begin{array}{cc} {∥\nabla e^{n}∥}_{0}\leq & \nu^{-1}N_{0}\left(\nu^{-1}m\alpha^{2}\left(\gamma^{-1}{∥f_{1}∥}_{-1}+D_{c}^{-1}{∥f_{2}∥}_{-1}\right)\right){∥\nabla e^{n-1}∥}_{0}\\ \; & +2\nu^{-1}N_{0}{∥\nabla e^{n-1}∥}_{0}(\nu^{-1}m\alpha^{2}\left(\gamma^{-1}{∥f_{1}∥}_{-1}+D_{c}^{-1}{∥f_{2}∥}_{-1}\right)\\ \; & +m\alpha^{2}\nu^{-1}(2\gamma^{-1}\nu^{-1}m\alpha^{2}\left(\gamma^{-1}N_{1}{∥f_{1}∥}_{-1}+D_{c}^{-1}N_{2}{∥f_{2}∥}_{-1}\right){∥\nabla \xi^{n-1}∥}_{0}\\ \; & +N_{1}\gamma^{-2}{∥f_{1}∥}_{-1}{∥\nabla e^{n-1}∥}_{0})\\ \; & +m\alpha^{2}\nu^{-1}(2D_{c}^{-1}\nu^{-1}m\alpha^{2}\left(\gamma^{-1}N_{1}{∥f_{1}∥}_{-1}+D_{c}^{-1}N_{2}{∥f_{2}∥}_{-1}\right){∥\nabla \delta^{n-1}∥}_{0}\\ \; & +N_{2}D_{c}^{-2}{∥f_{2}∥}_{-1}{∥\nabla e^{n-1}∥}_{0})\\ \leq & 3\nu^{-1}N_{0}{\left(3\sigma \right)}^{n-1}\nu^{-2}m^{2}\alpha^{4}{\left(\gamma^{-1}{∥f_{1}∥}_{-1}+D_{c}^{-1}{∥f_{2}∥}_{-1}\right)}^{2}\\ \; & +m\alpha^{2}\nu^{-1}(N_{1}\gamma^{-2}{∥f_{1}∥}_{-1}{\left(3\sigma \right)}^{n-1}\nu^{-1}m\alpha^{2}\left(\gamma^{-1}{∥f_{1}∥}_{-1}+D_{c}^{-1}{∥f_{2}∥}_{-1}\right)\\ \; & +2\gamma^{-1}\nu^{-1}m\alpha^{2}\left(\gamma^{-1}N_{1}{∥f_{1}∥}_{-1}+D_{c}^{-1}N_{2}{∥f_{2}∥}_{-1}\right){\left(3\sigma \right)}^{n-1}\gamma^{-1}{∥f_{1}∥}_{-1})\\ \; & +m\alpha^{2}\nu^{-1}(N_{2}D_{c}^{-2}{∥f_{2}∥}_{-1}{\left(3\sigma \right)}^{n-1}\nu^{-1}m\alpha^{2}\left(\gamma^{-1}{∥f_{1}∥}_{-1}+D_{c}^{-1}{∥f_{2}∥}_{-1}\right)\\ \; & +2D_{c}^{-1}\nu^{-1}m\alpha^{2}\left(\gamma^{-1}N_{1}{∥f_{1}∥}_{-1}+D_{c}^{-1}N_{2}{∥f_{2}∥}_{-1}\right){\left(3\sigma \right)}^{n-1}D_{c}^{-1}{∥f_{2}∥}_{-1})\\ \leq & {\left(3\sigma \right)}^{n}\nu^{-1}m\alpha^{2}\left(\gamma^{-1}{∥f_{1}∥}_{-1}+D_{c}^{-1}{∥f_{2}∥}_{-1}\right),\;\forall n\geq 1. \end{array}$$

Finally, combining the first equation of (38) with q=0 and the discrete inf-sup condition, the error estimate for the pressure can be stated as follows

$$\begin{array}{cc} {∥\eta^{n}∥}_{0}\leq & \beta^{-1}(\nu {∥\nabla e^{n}∥}_{0}+N_{0}{∥\nabla u_{h}^{n-1}∥}_{0}{∥\nabla e^{n-1}∥}_{0}+N_{0}{∥\nabla e^{n-1}∥}_{0}{∥\nabla u_{h}∥}_{0}\\ \; & +m\alpha^{2}\left({∥\nabla \xi^{n}∥}_{0}+{∥\nabla \delta^{n}∥}_{0}\right))\\ \leq & \beta^{-1}(\nu {\left(3\sigma \right)}^{n}\nu^{-1}m\alpha^{2}\left(\gamma^{-1}{∥f_{1}∥}_{-1}+D_{c}^{-1}{∥f_{2}∥}_{-1}\right)\\ \; & +N_{0}{\left(3\sigma \right)}^{n-1}\nu^{-1}m\alpha^{2}\left(\gamma^{-1}{∥f_{1}∥}_{-1}+D_{c}^{-1}{∥f_{2}∥}_{-1}\right)2\nu^{-1}m\alpha^{2}\left(\gamma^{-1}{∥f_{1}∥}_{-1}+D_{c}^{-1}{∥f_{2}∥}_{-1}\right)\\ \; & +N_{0}{\left(3\sigma \right)}^{n-1}\nu^{-1}m\alpha^{2}\left(\gamma^{-1}{∥f_{1}∥}_{-1}+D_{c}^{-1}{∥f_{2}∥}_{-1}\right)m\alpha^{2}\left(\gamma^{-1}{∥f_{1}∥}_{-1}+D_{c}^{-1}{∥f_{2}∥}_{-1}\right)\\ \; & +m\alpha^{2}{\left(3\sigma \right)}^{n}\left(\gamma^{-1}{∥f_{1}∥}_{-1}+D_{c}^{-1}{∥f_{2}∥}_{-1}\right))\\ \leq & 5\beta^{-1}{\left(3\sigma \right)}^{n}m\alpha^{2}\left(\gamma^{-1}{∥f_{1}∥}_{-1}+D_{c}^{-1}{∥f_{2}∥}_{-1}\right). \end{array}$$

□

## 5. Numerical Experiments

In this section, several numerical experiments are presented to compare these iterative methods for the considered equations. We use the public finite element software FreeFem++ [28].

### 5.1. An Analytical Solution Problem

For numerical implementations, the iterative tolerance is $1.0\times 10^{-5}$. The first issue to be considered here is to compare these iterative methods for the stationary double-diffusive natural convection in the case of $\Omega =\left[0,1\right]\times \left[0,1\right]\in R^{2}$, to reveal the relationship between the iterative methods and the viscosity. We consider the following exact solutions.

(42) $$\begin{array}{cc} p(x,y) & =\cos(\pi x)\cos(\pi y),\\ u_{1}\left(x,y\right) & =2\pi \sin^{2}\left(\pi x\right)\sin\left(\pi y\right)\cos\left(\pi y\right),\\ u_{2}\left(x,y\right) & =-2\pi \sin\left(\pi x\right)\sin^{2}\left(\pi y\right)\cos\left(\pi x\right),\\ T(x,y) & =u_{1}\left(x,y\right)+u_{2}\left(x,y\right),\\ C(x,y) & =u_{1}\left(x,y\right)-u_{2}\left(x,y\right). \end{array}$$

Set the Darcy number $D_{a}=1$, the thermal expansion coefficient $\beta_{T}=1$, the solutal expansion coefficient $\beta_{C}=1$, the heat diffusivity γ=1, the mass diffusivity $D_{c}=1$ and $u_{i}=0$, T=0, C=0 on ∂Ω,i=1,2. The forcing function $f_{i}$ can be calculated using (42), *i* = 1,2. We use a fixed value of mesh size $h=\frac{1}{64}$, and perform tests for the values of the viscosity coefficients going from ν=1 to $\nu =1.0\times 10^{-4}$.

We compare the numbers of iteration and the computational time in Table 1. This table shows that all iterative methods run well in the case of ν=1. When the viscosity number increases to $\nu =1.0\times 10^{-2}$, iterative method III is divergent. Finally, iterative methods II and III can not export the data with $\nu =1.0\times 10^{-4}$, iterative method I is still convergent. From a computational point of view, the calculation time of iterative method I and iterative method II is similar. However, iterative method II saves about 30% of calculation time compared iterative method III when ν=1. Iterative method II saves about 35% of calculation time compared with iterative method I when $\nu =1.0\times 10^{-2}$. We can conclude that iterative method III is the simplest method for a high viscosity number. The iterative method II is a fast and high accuracy method for a slightly lower viscosity number. Iterative method I is stable under uniqueness condition of weak solutions in the case of the lowest viscosity number. For three iterative methods, the relative error estimates are presented in Table 2, Table 3 and Table 4.

### 5.2. The Cavity Problem

**Figure 1:**
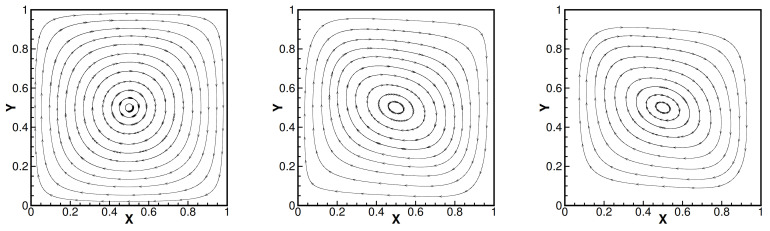

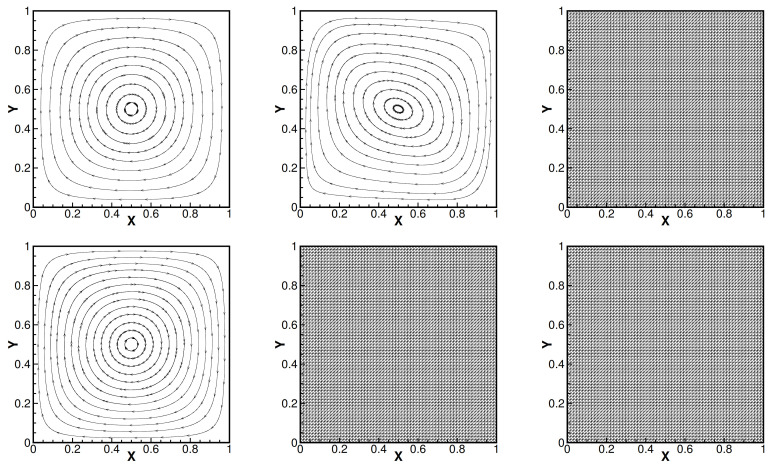
Velocity streamlines of iteration method I (the first line), iteration method II (the second line) and iteration method III (the third line) with different viscosity coefficients 1.0 (the first column), $1.0\times 10^{-3}$ (the second column) and $1.0\times 10^{-4}$ (the third column). $D_{a}=1$, $\beta_{T}=1$, $\beta_{C}=1$, γ=0.1, $D_{c}=0.01$.

In this numerical experiment, we assume that the boundary conditions satisfy [7,9]

(43) $$\begin{array}{cc} T=1,\;\;C=1,\;u=0\; & at\;x_{1}=0,\\ T=-1,\;C=-1,\;u=0\; & at\;x_{1}=1,\\ \frac{\partial T}{\partial n}=0,\;\frac{\partial C}{\partial n}=0,\;u=0\; & at\;x_{2}=0,\\ \frac{\partial T}{\partial n}=0,\;\frac{\partial C}{\partial n}=0,\;u=0\; & at\;x_{2}=1, \end{array}$$

and set $D_{a}=1$, $\beta_{T}=1$, $\beta_{C}=1$, γ=0.1, $D_{c}=0.01$, $f_{i}=0$ and the mesh size $h=\frac{1}{64}$, i=1,2. Moreover, the convergence tolerance is set to equal $1.0\times 10^{-6}$. The domain with its boundary conditions is illustrated in Figure 1. We present the velocity streamlines, the pressure isobars, the isotherms and the isoconcentration lines for different values of the viscosity coefficients ν=1.0, $\nu =1.0\times 10^{-3}$, $\nu =1.0\times 10^{-4}$.

**Figure 2:**
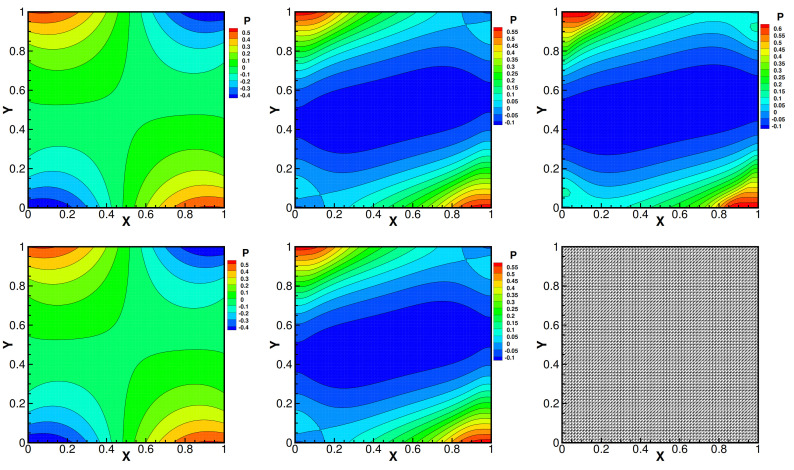

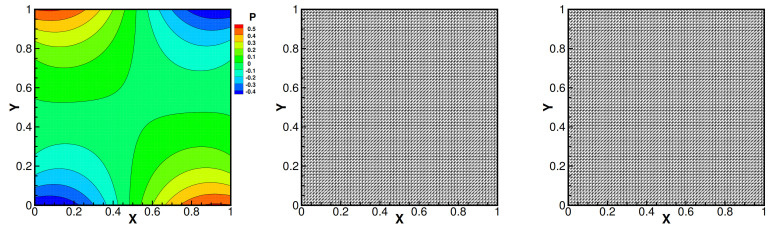
Pressure isobars of iteration method I (the first line), iteration method II (the second line) and iteration method III (the third line) with different viscosity coefficients 1.0 (the first column), $1.0\times 10^{-3}$ (the second column) and $1.0\times 10^{-4}$ (the third column). $D_{a}=1$, $\beta_{T}=1$, $\beta_{C}=1$, γ=0.1, $D_{c}=0.01$.

**Figure 3:**
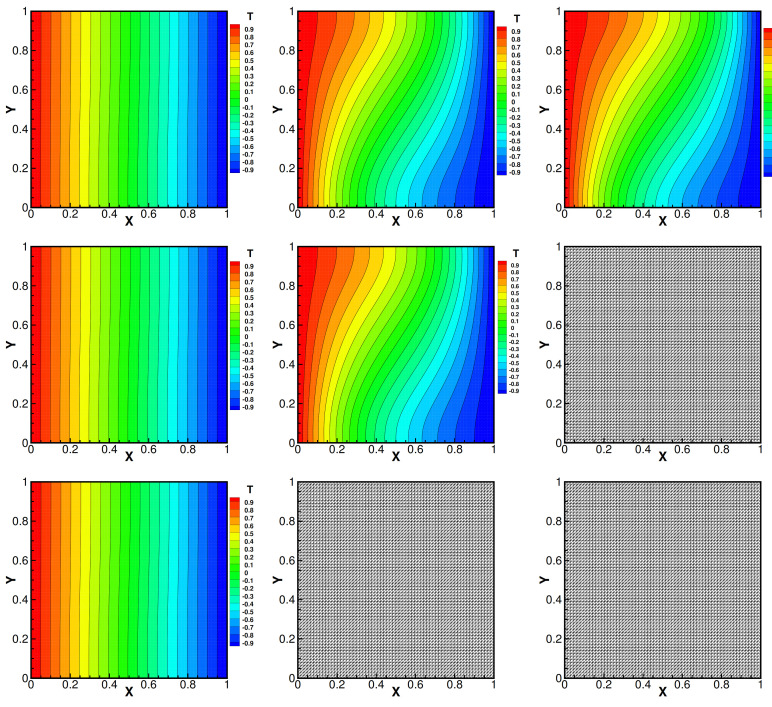
Isotherms of iteration method I (the first line), iteration method II (the second line) and iteration method III (the third line) with different viscosity coefficients 1.0 (the first column), $1.0\times 10^{-3}$ (the second column) and $1.0\times 10^{-4}$ (the third column). $D_{a}=1$, $\beta_{T}=1$, $\beta_{C}=1$, γ=0.1, $D_{c}=0.01$.

**Figure 4:**
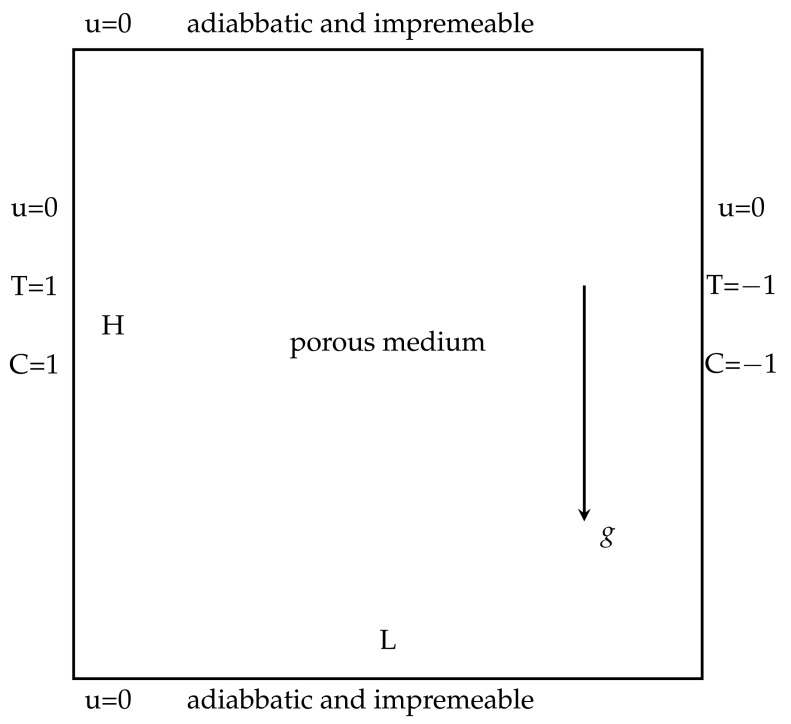
The computational domain with its boundary conditions.

**Figure 5:**
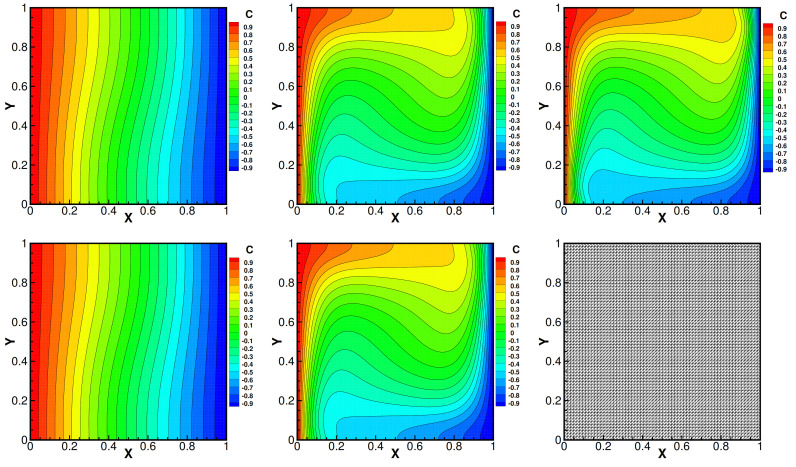

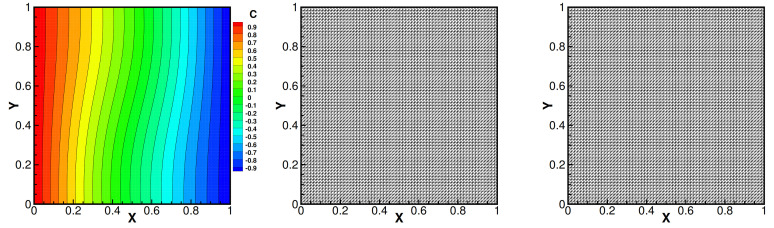
Isotherms of iteration method I (the first line), iteration method II (the second line), iteration method III (the third line) with different viscosity coefficients 1.0 (the first column), $1.0\times 10^{-3}$ (the second column) and $1.0\times 10^{-4}$ (the third column). $D_{a}=1$, $\beta_{T}=1$, $\beta_{C}=1$, γ=0.1, $D_{c}=0.01$.

Then, we show numerical velocity streamlines, isobars of pressure, isotherms, and isoconcentration lines obtained by three iterative methods with different viscosity numbers. We plot these results in Figure 2, Figure 3, Figure 4 and Figure 5. From these graphs, we obtain that the values of viscosity not only heavily impact on the velocity streamlines and the isobars, but also affect the isotherms and the isoconcentration lines. In fact, three iterations run well with ν=1.0. However, iterative method III cannot run with $\nu =1.0\times 10^{-3}$ while iterative method II cannot export the data with $\nu =1.0\times 10^{-4}$.

**Figure 6:**
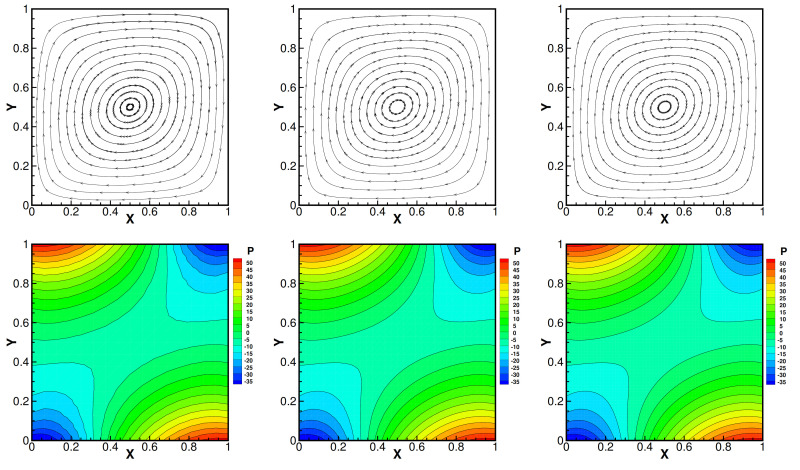
Velocity streamlines (the first line) and pressure isobars (the second line) of iteration method I with different mesh size $h=\frac{1}{16}$ (the first column), $h=\frac{1}{32}$ (the second column) and $h=\frac{1}{64}$ (the third column). ν=1, $D_{a}=0.01$, $\beta_{T}=100$, $\beta_{C}=100$, γ=0.1, $D_{c}=0.1$.

To consider the independency of mesh in a square cavity, we use iterative method I to calculate the model (1) under different mesh sizes. The results are presented in Figure 6. We can see that there is no difference in the calculation results under different mesh sizes, so we can verify the independence of the mesh size.

## 6. Conclusions

In conclusion, for solving stationary double-diffusive natural convection equations, three iterative methods have their own advantages under different viscosity numbers. In the case of $0<\sigma <\frac{1}{4}$, all methods can export data. Moreover, in the case of $\frac{1}{4}\leq \sigma <\frac{1}{3}$, iterative method I and II can run well. Finally, in the case of $\frac{1}{3}\leq \sigma <1$, only iterative method I can export data.

From the perspective of physical applications, these finite element iterative methods can be used to simulate different double-diffusive natural convection models, such as the aluminum oxide nanofluid natural convection heat transfer, the natural convection flow of a suspension containing nano-encapsulated. Furthermore, some different boundary conditions of these models with some different calculation areas should be considered, such as the T-geometry enclosure porous cavity, L-geometry cavity, and porous cavity.

## Figures and Tables

**Table 1:** CPU-time in second (iterative step) needed to reach the convergence tolerance.

Scheme	ν =1	$\nu =1.0\times 10^{-2}$	$\nu =1.0\times 10^{-4}$
I	50.696 (4)	174.857 (14)	424.661 (41)
II	49.432 (4)	112.317 (6)	—
III	78.703 (7)	—	—

**Table 2:** Comparison of three iterative methods using $P_{2}-P_{1}-P_{2}-P_{2}$ ($h=\frac{1}{64}$ and ν=1).

Scheme	$\frac{{∥\nabla (u-u_{h}^{n})∥}_{0}}{{∥\nabla u∥}_{0}}$	$\frac{{∥p-p_{h}^{n}∥}_{0}}{{∥p∥}_{0}}$	$\frac{{∥\nabla (T-T_{h}^{n})∥}_{0}}{{∥\nabla T∥}_{0}}$	$\frac{{∥\nabla (C-C_{h}^{n})∥}_{0}}{{∥\nabla C∥}_{0}}$
I	0.000717912	0.000206301	0.000359132	0.00094964
II	0.000717912	0.000206303	0.000359132	0.00094964
III	0.000717912	0.000206251	0.000359145	0.000949645

**Table 3:** Comparison of three iterative methods using $P_{2}-P_{1}-P_{2}-P_{2}$ ($h=\frac{1}{64}$ and $\nu =1.0\times 10^{-2}$).

Scheme	$\frac{{∥\nabla (u-u_{h}^{n})∥}_{0}}{{∥\nabla u∥}_{0}}$	$\frac{{∥p-p_{h}^{n}∥}_{0}}{{∥p∥}_{0}}$	$\frac{{∥\nabla (T-T_{h}^{n})∥}_{0}}{{∥\nabla T∥}_{0}}$	$\frac{{∥\nabla (C-C_{h}^{n})∥}_{0}}{{∥\nabla C∥}_{0}}$
I	0.000738137	0.000200965	0.000359132	0.00094964
II	0.000738136	0.00020096	0.000359132	0.00094964
III	—	—	—	—

**Table 4:** Comparison of three iterative methods using $P_{2}-P_{1}-P_{2}-P_{2}$ ($h=\frac{1}{64}$ and $\nu =1.0\times 10^{-4}$).

Scheme	$\frac{{∥\nabla (u-u_{h}^{n})∥}_{0}}{{∥\nabla u∥}_{0}}$	$\frac{{∥p-p_{h}^{n}∥}_{0}}{{∥p∥}_{0}}$	$\frac{{∥\nabla (T-T_{h}^{n})∥}_{0}}{{∥\nabla T∥}_{0}}$	$\frac{{∥\nabla (C-C_{h}^{n})∥}_{0}}{{∥\nabla C∥}_{0}}$
I	0.00759437	0.000203286	0.000359133	0.000949641
II	—	—	—	—
III	—	—	—	—

## Data Availability

Data sharing not applicable.

## Abbreviations

*a*	bilinear form	*w*	mapping difference
*A*	a mapping	*W*	temperature space
*c*	trilinear form	*x*	dimensionless coordinate
*C*	concentration	*X*	velocity space
$D_{a}$	Darcy number	*y*	dimensionless coordinate
$D_{c}$	mass diffusivity	Greeksymbols	
*e*	iterative error of velocity	$\beta_{T}$	thermal expansion coefficient
*f*	forcing function	$\beta_{C}$	solutal expansion coefficient
*g*	gravitational acceleration vector	β	positive constant
*h*	mesh size	γ	heat diffusivity
*H*	dual space	ψ	test function for temperature
*k*	iterative step	σ	uniqueness condition
*K*	triangular region	λ	constant[0,1]
*L*	Lebegue space	ν	viscosity
*m*	$m=\left|g\right|\max\{\left|\beta_{T}\right|,\left|\beta_{C}\right|\}$	α	Poincaré constant
*M*	pressure space	η	iterative error of pressure
*n*	iterative step	δ	iterative error of concentration
*N*	constant	ξ	iterative error of temperature
*p*	fluid pressure	Subscript	
*P*	polynomial	*i*	1,2
*q*	test function for pressure	h	finite element discretization
*Q*	concentration space		
*s*	test function for concentration		
*T*	temperature		
*u*	velocity field		
*v*	test function for velocity		
*V*	subspace of the velocity space		
